# Metabolic Trajectories Following Contrasting Prudent and Western Diets from Food Provisions: Identifying Robust Biomarkers of Short-Term Changes in Habitual Diet

**DOI:** 10.3390/nu11102407

**Published:** 2019-10-09

**Authors:** Nadine Wellington, Meera Shanmuganathan, Russell J. de Souza, Michael A. Zulyniak, Sandi Azab, Jonathon Bloomfield, Alicia Mell, Ritchie Ly, Dipika Desai, Sonia S. Anand, Philip Britz-McKibbin

**Affiliations:** 1 Department of Chemical and Chemical Biology, McMaster University, Hamilton, ON L8S 4M1, Canada; 2 Department of Medicine, McMaster University, Hamilton, ON L8S 4K1, Canada; 3 Department of Health Research Methods, Evidence, and Impact, McMaster University, Hamilton, ON L8S 4K1, Canada; 4 Population Health Research Institute, Hamilton, ON L8L 2X2, Canada; 5 School of Food Science and Nutrition, University of Leeds, LS2 9JT Leeds, UK

**Keywords:** metabolomics, metabolite profiling, Prudent diet, Western diet, food provisions, diet records, nutritional epidemiology, mass spectrometry

## Abstract

A large body of evidence has linked unhealthy eating patterns with an alarming increase in obesity and chronic disease worldwide. However, existing methods of assessing dietary intake in nutritional epidemiology rely on food frequency questionnaires or dietary records that are prone to bias and selective reporting. Herein, metabolic phenotyping was performed on 42 healthy participants from the Diet and Gene Intervention (DIGEST) pilot study, a parallel two-arm randomized clinical trial that provided complete diets to all participants. Matching single-spot urine and fasting plasma specimens were collected at baseline, and then following two weeks of either a Prudent or Western diet with a weight-maintaining menu plan designed by a dietician. Targeted and nontargeted metabolite profiling was conducted using three complementary analytical platforms, where 80 plasma metabolites and 84 creatinine-normalized urinary metabolites were reliably measured (CV < 30%) in the majority of participants (>75%) after implementing a rigorous data workflow for metabolite authentication with stringent quality control. We classified a panel of metabolites with distinctive trajectories following two weeks of food provisions when using complementary univariate and multivariate statistical models. Unknown metabolites associated with contrasting dietary patterns were identified with high-resolution MS/MS, as well as co-elution after spiking with authentic standards if available. Overall, 3-methylhistidine and proline betaine concentrations increased in both plasma and urine samples after participants were assigned a Prudent diet (*q* < 0.05) with a corresponding decrease in the Western diet group. Similarly, creatinine-normalized urinary imidazole propionate, hydroxypipecolic acid, dihydroxybenzoic acid, and enterolactone glucuronide, as well as plasma ketoleucine and ketovaline increased with a Prudent diet (*p* < 0.05) after adjustments for age, sex, and BMI. In contrast, plasma myristic acid, linoelaidic acid, linoleic acid, α-linoleic acid, pentadecanoic acid, alanine, proline, carnitine, and deoxycarnitine, as well as urinary acesulfame K increased among participants following a Western diet. Most metabolites were also correlated (*r* > ± 0.30, *p* < 0.05) to changes in the average intake of specific nutrients from self-reported diet records reflecting good adherence to assigned food provisions. Our study revealed robust biomarkers sensitive to short-term changes in habitual diet, which is needed for accurate monitoring of healthy eating patterns in free-living populations, and evidence-based public health policies for chronic disease prevention.

## 1. Introduction

A global epidemic of obesity and chronic non-communicable diseases threaten to reduce life expectancy and impose a severe burden on public health [1,2]. Diet and lifestyle are two key modifiable determinants of human health of particular importance for risk of cardiovascular disease (CVD), type 2 diabetes, and some cancers [3]. CVD remains the leading cause of death globally [4], which has been associated with a Western diet. Contemporary Western diets rich in *trans* fats, processed foods, and red meat, including regular consumption of sweetened beverages and high glycemic index foods lacking adequate fiber, have been strongly linked to chronic inflammation, and metabolic syndrome [5]. These deleterious eating patterns increasingly impact cardiometabolic health across the lifespan [6]. In contrast, a Prudent diet and analogous health-promoting diets (e.g., DASH, Mediterranean, Nordic) that include a greater intake of fruits and vegetables, lean meats, and whole grains, reduce blood lipids, improve blood sugar homeostasis, and lower blood pressure [7,8]. However, there is an urgent need for more accurate dietary assessment tools for the validation of nutritional policies that are effective for chronic disease prevention on a population level, unlike the traditional diet-heart hypothesis [9].

Nutritional epidemiologists face unique challenges in light of the highly complex chemical composition of foods, whose physiological effects are often confounded by interactions of diet with genes, lifestyle, microbiome, and other environmental exposures [10]. To date, observational studies in nutrition mainly rely on self-reported measures of dietary intake, including methods of recall (e.g., food frequency questionnaires, 24 h dietary recall) or real-time recording (e.g., food diaries) that are prone to bias and selective reporting [11]. Alternatively, targeted assays exist for measuring energy expenditure (e.g., doubly-labeled water), as well as specific macronutrients (e.g., protein), electrolytes (e.g., sodium) and micronutrients (e.g., vitamin D) with established reference ranges associated with nutritional status and/or chronic disease risk. However, these methods are not routinely applied in large-scale human studies due to major cost barriers while representing only a small fraction of total food exposures [12,13]. In this context, new advances in high throughput metabolomics offer a holistic approach for measuring complex dietary patterns in lieu of specific nutrients in human biofluids, such as blood and urine [14]. Recent metabolomic studies have identified dietary biomarkers [15,16] to monitor for dietary adherence, as well as validate or correct standard dietary assessment methods used in nutritional epidemiology [17,18,19,20,21]. However, few dietary biomarkers are unique to specific foods, nor adequately validated as quantitative measures of recent, or habitual food intake in well-controlled randomized clinical trials [22,23,24].

Herein, metabolic phenotyping of matching plasma and urine specimens were analyzed from healthy participants from the Diet and Gene Intervention Study (DIGEST), which was a randomized controlled trial to explore the short-term effects of a Prudent diet on CVD risk factors, where individuals were provided all foods to prepare at home [25]. A modest reduction in systolic and diastolic blood pressure, and total cholesterol was reported for participants following a Prudent diet after two weeks as compared to a Western diet; however, dietary adherence relied on participant self-reporting, and food preparation methods were not standardized likely contributing to variability in treatment responses [25]. In this work, we sought to identify specific metabolic trajectories in plasma and urine that may serve as responsive biomarkers reflecting short-term changes in habitual diet, which were measured in free-living individuals outside of a dedicated metabolic ward or hospital stay. These dietary biomarkers not only confirmed good adherence to assigned food provisions, but were also associated with healthy eating patterns indicative of a Prudent diet [26], as compared to a Western diet that increases overall risk for CVD [27].

## 2. Experimental

### 2.1. Study Design, Participant Eligibility and Dietary Self-reporting

DIGEST was a two-arm, parallel unblinded study to compare the effects of two weeks of a Prudent diet as compared to a Western diet on CVD risk factors, and gene expression. Healthy participants were recruited using flyers, and self-referral methods from McMaster University and the surrounding areas. Exclusion criteria were an unwillingness to eat an assigned diet, or serious disease or illness, including cardiometabolic and neurodegenerative disorders, with the full cohort selection criteria described elsewhere [25]. A subset of 42 participants from DIGEST with paired urine and serum samples available were selected for targeted and nontargeted metabolomics using three complementary instrumental platforms, where participants had also completed self-reported dietary records at baseline, and each week for two weeks following assigned food provisions. All participants from DIGEST picked up their food allotment each week at the grocery store, during clinic visits, or food provisions were delivered to their home by volunteers [25]. No detailed information was collected on food preparation methods at home involving non-packaged foods; however all participants were requested to maintain their normal lifestyle habits during the intervention period (e.g., physical activity, cooking methods). A Harris-Benedict equation plus an activity factor was used to estimate participant’s energy intake, compared this against their 7-day diet record, and assigned the appropriate diet plan at one of nine energy levels (from 1600 through 3200 kcal) designed to maintain weight during the study period. After the first week of intervention, participants were re-weighed and the food for the second week was maintained, increased or decreased (using groups of 100 kcal snacks designed for each diet) as necessary to maintain baseline weight. In order to maximize treatment effects in this short-term pilot study, participants were assigned into two parallel arms of contrasting diets, namely a Western diet reflecting a typical Canadian macronutrient profile with higher intake of processed foods (e.g., burgers, fried chicken, cereals, processed cheeses), and a Prudent diet based around minimally processed foods of lean protein (e.g., poultry, fish, legumes), whole grains, and a high amount of fresh fruits and vegetables [25].

Briefly, one week prior to beginning the intervention diet, all DIGEST participants attended a pre-study visit, where they were instructed by staff on how to complete a prospective 7-day food diary. An instruction sheet and booklet were also provided. Participants were provided with a blank diary and asked to record each item consumed at each eating occasion (e.g., food name, amount, method of cooking). Common household measures were used for food and drink (e.g., cups, tablespoons). For composite foods that could not be readily split, such as a fruit pie or meat casserole, participants were asked to provide recipe details. Foods eaten away from home were recorded in as much detail as possible (e.g., restaurant name, item). Study staff reviewed the food diary with each participant on the day of the baseline visit. At the beginning of each week of the intervention period, each participant was provided with a 7-day meal plan that listed the exact food items and quantities they were to eat. As each food was eaten, participants were instructed to check that item off the list. Participants also recorded any additional items consumed that were not on the checklist. This checklist was returned at the end of each of the two weeks during the intervention, and study staff reviewed this with each participant. The pre-study diets were entered into, and analyzed using The Food Processor^®^ (ESHA Research Inc., Salem, OR, USA) for micro- and macronutrients, and servings of fruits and vegetables. A diet quality index score was assigned for each participant at baseline, and an average over the two week intervention period as follows; one "Prudent" point was assigned for each of the following criteria: polysaturated:saturated fat ratio (poly:sat) > 1.0, saturated fat < 7%, total fiber > 28 g/day, fruits + vegetables > 5 servings/day, and potassium > 3500 mg/day. The maximum score was 5. Similarly, one "Western" point was assigned for each of the following criteria: poly:sat < 0.5, saturated fat > 16%, total fiber < 9 g/day, fruits + vegetables < 5 servings/day, and potassium < 3500 mg/day. The maximum score was 5. DIGEST participants were classified as "predominantly" Prudent if the Prudent score minus the Western score was ≥ 2, or they were classified as "predominantly" Western if the Western score minus the Prudent score was ≥ 2; however, based on these criteria, few participants had Prudent diet characteristics (14%; 6 out of 42) with most having Western eating habits at baseline (86%, 36 out of 42). Dietary adherence was a measure of the % of the prescribed foods that they reported eating based on the foods they checked-off from a menu list that they consumed, which was > 95% for both treatment arms. A total of 20 micro- and macronutrient categories (from over 120) from self-reporting dietary records were different (*q* < 0.05; Bonferroni adjustment) between assigned Prudent and Western diets among DIGEST participants, which were subsequently correlated with top-ranked plasma and urinary metabolites when classifying putative biomarkers of contrasting diets.

### 2.2. Chemicals and Reagents

Ultra HPLC grade LC-MS solvents (water, methanol, acetonitrile) obtained from Caledon Laboratories Ltd. (Georgetown, ON, Canada) were used to prepare all buffer and sheath liquid solutions. Proline betaine was purchased from Toronto Research Chemicals Inc. (Toronto, ON, Canada). All other chemicals were obtained from Sigma-Aldrich Inc. (St. Louis, MO, USA).

### 2.3. Nontargeted Metabolite Profiling of Plasma and Urine by MSI-CE-MS

Fasting plasma (EDTA) samples together with matching single-spot urine samples were collected from all DIGEST participants during clinic visits on day 1 and day 14, which were then stored at −80 °C [25]. Multisegment injection-capillary electrophoresis-mass spectrometry (MSI-CE-MS) was the major platform used for nontargeted profiling of polar/ionic metabolites from both plasma and urine samples [28], which was performed on an Agilent G7100A CE (Agilent Technologies Inc., Mississauga, ON, Canada) equipped with a coaxial sheath liquid (Dual AJS) Jetstream electrospray ion source coupled to an Agilent 6230 TOF-MS system. All separations used uncoated fused-silica capillaries (Polymicro Technologies Inc., Phoenix, AZ, USA) with a total length of 120 cm and an inner diameter of 50 μm. About 7 mm of polyimide coating was removed from both distal ends to avoid sample carry-over and prevent polyimide swelling from contact with organic solvent [29]. The background electrolyte (BGE) consisted of 1 M formic acid with 15% volume acetonitrile (pH 1.8) under positive ion mode, and 50 mM ammonium bicarbonate (pH 8.5) under negative ion mode for characterization of the ionic metabolome, including cationic and anionic metabolites from matching plasma and urine specimens, respectively. All CE separations were performed under normal polarity with an applied voltage of 30 kV at 25 °C. However, a pressure gradient of 2 mbar/min from 0 to 40 min was used for MSI-CE-MS analyses under negative ion mode conditions to shorten analysis times for highly charged anionic metabolites (e.g., citrate). The TOF-MS system was operated with full-scan data acquisition over a mass range of *m*/*z* 50-1700 and an acquisition rate of 500 ms/spectrum. The sheath liquid was comprised of 60% volume MeOH with 0.1% volume formic acid for positive ion mode, and 50% volume MeOH for negative ion mode. The ESI conditions were Vcap = 2000 V, nozzle voltage = 2000 V, nebulizer gas = 10 psi, sheath gas = 3.5 L/min at 195 °C, drying gas 8 L/min at 300 °C, and the MS voltage settings were fragmentor = 120 V, skimmer = 65 V and Oct1 RF = 750 V.

Thawed plasma (50 μL) on ice was diluted four-fold with deionized water containing 20 μM 3-chloro-*L*-tyrosine (Cl-Tyr), 20 μM 4-fluoro-*L*-phenylalanine (F-Phe), and 20 μM naphthalene monosulfonic acid (NMS) as internal/recovery standards. Diluted plasma samples were vortexed for 30 s and then plasma protein was filtered via ultracentrifugation using a 3 kDa MWCO Nanosep centrifugal device (Pall Life Sciences, Port Washington, NY, USA) at 14,000 *g* for 15 min prior to analysis. All filters were initially pre-washed in deionized water prior to plasma addition. Frozen urine was thawed slowly on ice, vortexed for 30 s, and particulate matter was sedimented by centrifugation at 14,000 *g* for 5 min. All urine samples were then diluted five-fold in deionized water containing Cl-Tyr, F-Phe and NMS as internal/recovery standards (20 μM). A seven sample serial injection format was used in MSI-CE-MS [30,31,32] consisting of a serial injection of six individual plasma filtrate or urine samples together with a pooled quality control (QC) within each experimental run; the latter sample was used to assess technical variance while also allowing for robust batch correction due to long-term signal drift in ESI-MS [31,33]. Multiplexed electrophoretic separations in MSI-CE-MS was performed by programming a hydrodynamic injection sequence with each sample (5 s at 100 mbar) alternating with a BGE spacer (40 s at 100 mbar) prior to voltage application as described elsewhere [30,31,32]. Nontargeted metabolite profiling of plasma or urine specimens by MSI-CE-MS was conducted by pairing together matching baseline and post-treatment samples from three DIGEST participants together with a QC in a randomized injection position for each run. Different serial sample injection configurations in MSI-CE-MS were also applied for rigorous authentication of metabolites using a dilution trend filter, as well as the acquisition of calibration curves for metabolite quantification [30,31,32]. Briefly, authentic metabolites were defined by their unique accurate mass, and relative migration time (*m*/*z*:RMT) under positive (+) or negative (−) ion mode detection, after filtering out spurious signals, background ions, redundant adducts, in-source fragments, and isotopic features that comprise the majority of signals in MS-based metabolomics [33]. Additionally, only frequently detected plasma or urinary metabolites measured in the majority of samples from DIGEST participants (> 75%) with acceptable technical precision based on repeated analysis of QC samples (mean CV < 30%) were included in the final data matrix as a way to reduce false discoveries and data over-fitting [34]. Missing (i.e., zero) data below method detection limits were replaced with a minimum value corresponding to half of the lowest response measured for a given metabolite in all samples analyzed. Overall, 70 and 50 authentic polar/ionic metabolites were measured consistently in the majority of urine and plasma samples by MSI-CE-MS, respectively including unknown ions, and recovery standards that fully satisfied our multi-level metabolomics selection criteria, and rigorous filtering procedure. Also, creatinine concentrations were measured by MSI-CE-MS, which were used to reduce biological variance and correct for differences in hydration status for single-spot urine samples analyzed in this study.

### 2.4. Unknown Metabolite Identification by MS/MS

High-resolution tandem mass spectrometry (MS/MS) was employed for structural elucidation of unknown metabolites of biological significance from pooled QC samples prepared in this study. Collisional–induced dissociation (CID) experiments were performed on an Agilent G7100A CE system with a coaxial sheath liquid Jetstream electrospray ion source connected to an Agilent 6500 iFunnel QTOF instrument. Metabolite identification in this work adopted reporting standards recommended from the Metabolomics Standards Initiative [35], including unambiguous identification (level 1) that was confirmed with matching MS/MS spectra, and co-migration with an authentic standard acquired on the same instrument. Additionally, tentative/probable identification (level 2) was achieved by comparison of MS/MS spectra from public databases or published literature, or partial annotation of MS/MS spectra guided by in silico software tools with metabolite class (level 3). Otherwise, compounds with unknown chemical structures were characterized by their most likely molecular formula (level 4); the latter case typically occurred for low abundance metabolites that had inadequate responses for their precursor ion when using MS/MS. Also, the electromigration behavior of polar/ionic metabolites (i.e., RMT) provided useful qualitative information that complemented MS/MS when selecting among several isobaric or isomeric candidate ions. MS/MS spectra were acquired from pooled plasma or urine samples that were injected hydrodynamically using a conventional single sample injection plug at 50 mbar for 10 s followed by 5 s with BGE. Precursor ions were selected for CID experiments at different collision energies (e.g., 10, 15, 20 or 40 V). Mirror plots depicting MS/MS spectra for unknown metabolites in pooled samples were acquired under an optimal collision energy, and then compared to their respective authentic reference standard (if available) under the same experimental conditions, which were generated using the “InterpretMSSpectrum” R Package. Otherwise, MS/MS spectra were annotated based on their characteristic product ions, and neutral mass losses for *de novo* structural elucidation (level 2 or 3), which was guided by in silico MS/MS spectra generated by CFM-ID [36]. Whenever possible, experimental MS/MS spectra were also compared to entries from curated/open-access public repositories (HMDB, http://www.hmdb.ca), or published in literature. The exact stereochemistry was uncertain for a subset of dietary biomarkers in plasma or urine if authentic standards were lacking. MS/MS spectra for five urinary metabolites associated with a Prudent diet in this work were also deposited onto HMDB.

### 2.5. Total Plasma Fatty Acid Determination by GC-MS

A GC-MS method was used for targeted analysis of 26 total (hydrolyzed) fatty acids (FA) and their isomers from plasma extracts on an Agilent 6890 GC coupled to an Agilent 5973 single quadrupole mass spectrometer with electron impact ionization (EI) with minor modifications [37]. Total hydrolyzed plasma FA were analyzed by GC-MS as their methyl ester derivatives (FAME) using *N*-methyl-*N*-(trimethylsilyl)trifluoroacetamide (MSTFA) (≥98.5%). Isotopically-labeled myristic acid-d27 (98%), stearic acid-d35 (98%) and pyrene-d10 (98%) were obtained from Cambridge Isotope Laboratories (Tewksbury, MA, USA). HPLC grade chloroform (≥99.5%), methanol (99.8%), hexanes (≥99.5%) and Ultra LC-MS grade water were purchased from Caledon Laboratories Ltd. (Georgetown, ON, Canada). The antioxidant, butylated hydroxytoluene (BHT, 490 µL) was added to all FA calibrant solutions prepared in methanol to prevent autooxidation. Briefly, a 10 µL aliquot of thawed plasma was mixed with 5 µL of a 1.0 mg/mL C18:0-d35 recovery standard. Concentrated sulfuric acid (10 µL) was added as a transesterification catalyst and vortexed for 2 min before incubation for 4 h at 80 °C to produce FAME for improved volatility. Cooled samples were then mixed with 500 µL of 9 g/L NaCl and 200 µL hexanes and vortexed for 2 min prior to centrifugation at 1400 *g* at 4 °C for 5 min. GC inserts were prepared with 45 µL of hexane supernatant with 5 µL of internal standard pyrene-d10 and vortexed for 2 min before injection. Total plasma FAME were resolved on a Supleco SP-2380 column (30 m × 0.25 mm × 0.20 µm) using an optimal temperature program within 30 min. Samples were injected in 1.0 µL volumes using a splitless injector held at 250 °C, the carrier gas was helium at 1.0 mL/min and the transfer line was held at 270 °C. Pooled QC and blank extracts were analyzed together with each batch of 8 to 10 randomized plasma samples when using GC-MS in order to assess technical precision and monitor for background contamination. A temperature program used for resolution of major FAME from plasma hydrolysates comprised of a temperature gradient of 20 °C/min starting from 2 min at 80 °C until 20 min at 160 °C, which was further increased to 190 °C for 3 min prior to elution at 300 °C for 5 min with a total run time of 28 min. In most cases, FAME were quantified in GC-MS based on the integration of the relative response ratio of their [M-15]^+^ fragment ion relative to pyrene-d10 as internal standard when using single ion monitoring (SIM) mode detection. Calibrations curves from FAME standards were used for FA quantification, as well as their identification when comparing their characteristic EI-MS spectra (70 eV) and elution times.

### 2.6. Targeted Urinary Electrolyte Analysis

Targeted analysis of 8 inorganic/involatile electrolytes in urine was performed using two complementary CE-UV methods adapted from Nori de Macedo et al. [38] and Saoi et al. [32] for anionic (e.g., iodide) and cationic (e.g., sodium) electrolytes, respectively. First, analysis of major cationic electrolytes was performed on frozen urine samples that were thawed, vortexed for 30 s and centrifuged at 14,000 *g* for 5 min. An aliquot of the urine supernatant was diluted five-fold with deionized water containing 0.5 mM lithium as an internal standard. Samples were analyzed on an Agilent G7100A CE system with UV photodiode array detection with indirect absorbance detection at 214 nm. All samples were injected hydrodynamically for 10 s (at 35 mbar) and separation was performed under normal polarity at 30 kV at 25 °C using a 50 µm inner diameter capillary with 60 cm total length. The background electrolyte (BGE) was 5 mM formic acid containing 12.5 mM creatinine, and 4 mM 18-crown-6 ether at pH 4.0 (adjusted with 1 M sulfuric acid). In this case, ammonium (NH_4_^+^), sodium (Na^+^), potassium (K^+^), calcium (Ca^2+^), and magnesium (Mg^2+^) were analyzed in urine samples [32]. Additionally, all urine samples were analyzed using a complementary CE assay for UV-absorbing inorganic anions, including nitrate (NO_3_^−^), iodide (I^−^), and thiocyanate (SCN^−^) [38]. In this case, the BGE was comprised of 180 mM lithium hydroxide, 180 mM phosphoric acid, and 10 mM α-cyclodextrin (α-CD) at pH 3 (adjusted with 1 M phosphoric acid), where 1,5-naphthalene disulfonic acid (NDS) was used as an internal standard. Samples were injected hydrodynamically for 80 s at 0.5 psi and analyzed at 25 °C under a reversed polarity at −18 kV with UV absorbance detection at 226 nm (288 nm for NDS). In both CE-UV assays, a pooled QC was measured intermittently after every batch of six urine samples.

### 2.7. Data Preprocessing and Statistical Analysis

All MSI-CE-MS data were integrated and analyzed using Agilent MassHunter Qualitative Analysis B.07.00 and Microsoft Excel and Igor (Wavemetrics Inc., Lake Oswego, OR, USA). In all cases, the integrated ion response (i.e., peak area) for each metabolite was normalized to an internal standard, Cl-Tyr migrating from the same sample by MSI-CE-MS, whereas F-Phe was used as a recovery standard to monitor for long-term technical precision in control charts. Also, a batch-correction algorithm was applied to creatinine-normalized urine metabolomic data to adjust for long-term signal drift in ESI-MS during data acquisition as outlined in a recent work [31]. This algorithm is based on an empirical Bayesian framework [39], which takes advantage of QC samples included in each serial injection run when using MSI-CE-MS, including batch order and injection sequence information. However, the batch-correction did not provide any significant improvement in the overall technical precision of plasma metabolome data and thus was not applied in this case. All non-batch (plasma) and batch-corrected (creatinine normalized urine) metabolomic data was pre-processed using generalized *log*-transformation, and autoscaling prior to multivariate statistical analysis using MetaboAnalyst 4.0 (www.metaboanalyst.ca) [40], including volcano plots, principal component analysis (PCA), hierarchical cluster analysis (HCA)/2D heat maps, receiver operating characteristic (ROC) curves, orthogonal partial least squares-discriminant analysis (OPLS-DA), as well as multivariate empirical Bayes analysis (MEBA) of variance; the latter method is optimal for analyzing time-series data [41] relevant to identifying metabolic trajectories reflective of contrasting diets. To validate each OPLS-DA model, cross-validation and permutation testing (*n* = 1000) on paired metabolome data sets (i.e., ratio of metabolite response based on assigned diet relative to baseline diet for each subject) following *glog* transformation and autoscaling, whereas Hotelling’s *T*-squared distribution using MEBA was performed on *glog*-transformed metabolomic time series data for DIGEST participants at baseline and following 2 weeks of food provisions. These complementary statistical approaches were used for unsupervised data exploration to inspect overall trends in reduced dimensionality, as well as supervised data analysis for ranking metabolites modulated by contrasting diets without adjustments for covariates. Additionally, normality tests, partial Pearson correlation analysis, and mixed model ANOVA were performed on top-ranked dietary biomarker candidates using the Statistical Package for the Social Sciences (SPSS, version 18.0). In this case, a partial listwise Pearson correlation analysis of lead plasma and creatinine-normalized metabolite responses to 20 major nutrient categories from self-reported food records from DIGEST participants (*n* = 42) were adjusted for age, sex, and post-intervention BMI. Only metabolites that had a correlation coefficient of *r* > ±0.300 and *p* < 0.05 for 2 or more nutrient categories were considered as significant in this work. A repeat measures general linear mixed ANOVA model was also performed with the number of levels set at 2 for the repeat sampling (i.e., time; baseline diet, and two weeks after assigned diet) while setting the intervention diet (i.e., treatment arm; P-W, and W-P) as the between-subject factor with age, sex, and post-intervention BMI as potential covariates. Overall, plasma and urine metabolites that satisfied MEBA and/or mixed ANOVA models, as well as partial correlation analysis to more than two nutrient categories from diet records were defined as robust dietary biomarkers in our study.

## 3. Results

### 3.1. Study Design, Baseline Habitual Diet and Metabolomics Workflow

**Figure 1:**
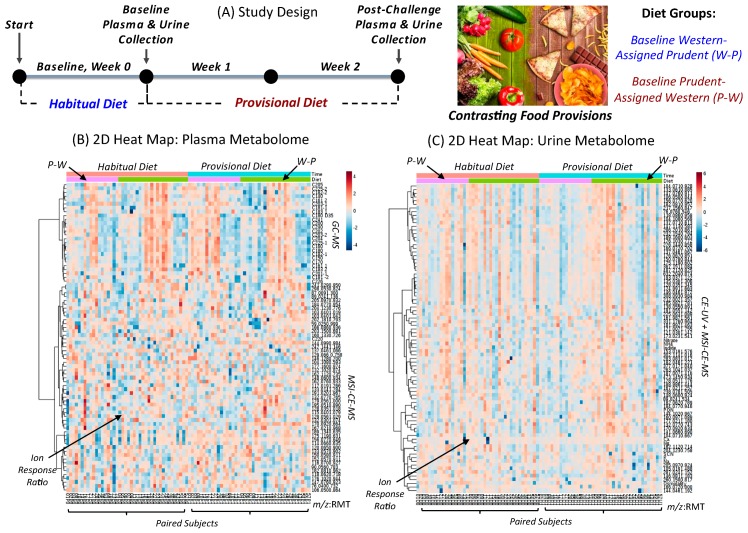
(**A**) Overview of study design in this parallel two-arm dietary intervention study involving participants (*n* = 42) from the Diet and Gene Interaction Study (DIGEST) who were assigned a Prudent or Western diet with matching urine and plasma collected at baseline, and two weeks post-intervention. (**B**) A 2D heat map with hierarchical cluster analysis (HCA) of the plasma metabolome, including nontargeted analysis of polar/ionic metabolites by MSI-CE-MS, and total fatty acids by GC-MS. (**C**) A 2D heat map with HCA of the urine metabolome, including nontargeted analysis of polar/ionic metabolites, and targeted electrolytes by CE with UV detection. A generalized *log* transformation and autoscaling were performed on metabolomic datasets together with batch-correction and creatinine normalization for single-spot urine specimens. Participants classified as having a predominate Western diet at baseline who were then assigned a Prudent diet are designated as “W-P” (*n* = 24), whereas “P-W” (*n* = 18) refers to participants who had a lower Western diet score at baseline, but were assigned a Western diet during the intervention period.

The DIGEST pilot study was a two-arm parallel dietary intervention involving healthy/non-smoking participants recruited from the local community as described elsewhere [25]. A CONCERT diagram summarizes eligibility criteria (Figure S1), where all participants completed a seven-day diet record and then were randomly allocated to eat a weight-maintaining Prudent or Western diet over two weeks. Participants (*n* = 42) with contrasting habitual diets were selected in this unblinded metabolomics study based on the availability of matching plasma and urine samples with complete diet records as depicted in Figure 1A. There were more women (64%) recruited than men, however there were no differences in age (mean age of 47 years ranging from 20 to 69 years), body composition (mean BMI of 27 kg/m^2^ with 26% defined as obese) and average caloric intake (mean of 1940 kcal/day) between assigned diet groups, and most participants were Caucasian (78%) with no self-identified tobacco smokers (Table S1). Also, no differences in baseline blood lipids, fasting glucose, inflammatory biomarkers, and blood pressure were evident between the two treatment arms. Since Prudent diet scores (<2) were low for this cohort overall (Table S1), 18 participants were classified as having a “Prudent-like” diet at baseline, but this was due to their lower average Western diet score (2.61 ± 0.69); this sub-group was randomized to an assigned Western diet (referred to as P-W) in this study. In contrast, 24 participants, having a predominate Western diet at baseline due to their higher average Western diet score (3.42 ± 0.93), were randomized to a Prudent diet (referred to as W-P). Importantly, there were no major differences in plasma or urine metabolic phenotypes measured at baseline, reflecting similar habitual dietary patterns for DIGEST participants in both treatment arms.

**Figure 2:**
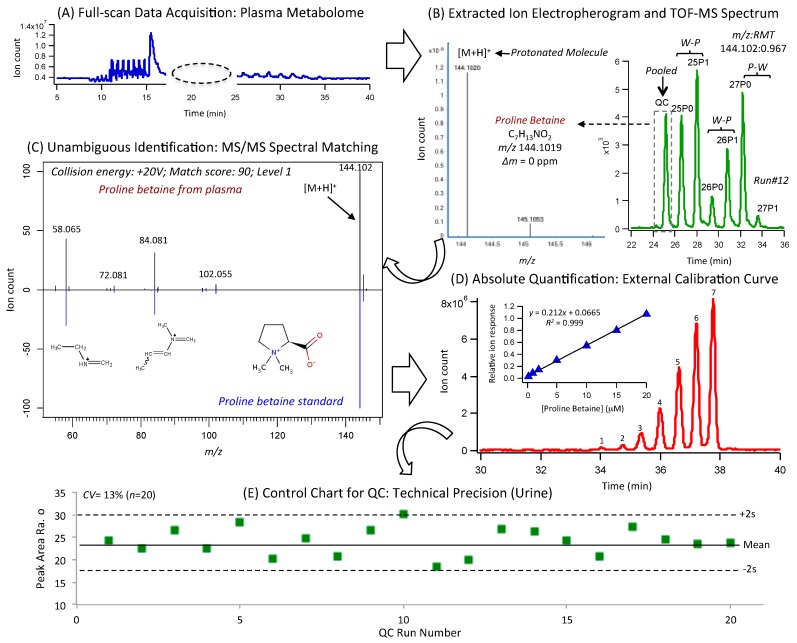
Summary of the metabolomics data workflow in MSI-CE-MS for the identification and quantification of biomarkers associated with a Prudent or Western diet. (**A**) A total ion electropherogram for plasma is shown with full-scan data acquisition under positive ion mode, whereas (**B**) an extracted ion electropherogram for an authentic unknown cation (i.e., [M+H]^+^) is annotated by its *m*/*z*:RMT based on serial injection of seven plasma samples within a single run. Each run is comprised three pairs of samples collected from DIGEST participants (i.e., baseline diet, and two weeks after food provisions) together with a QC for assessing technical precision, and correcting long-term signal drift. High-resolution MS spectra allows for determination of the most likely molecular formula, and (**C**) MS/MS spectra enable structural elucidation of the ion when compared to an authentic standard for ProBet. (**D**) Quantification of ProBet is performed by external calibration when using an internal standard (Cl-Tyr) for data normalization by MSI-CE-MS. (**E**) A control chart for ProBet from repeated measurements of QC samples in every MSI-CE-MS run demonstrates acceptable technical precision (CV = 13%, *n* = 20) over 3 days of analysis.

Figure 1B,C depict 2D heat maps for matching plasma and urine metabolomes measured from DIGEST participants (*n* = 42) at baseline and following two weeks of assigned food provisions. A total of 80 serum metabolites and 84 urinary metabolites were reliably measured (CV < 30%) in the majority of participants (> 75%) when using a validated data workflow for non-targeted metabolite profiling with stringent QC [30,31,32]. A rigorous approach to metabolite authentication was implemented to reject spurious, redundant, and background ions that comprise the majority of molecular features detected in ESI-MS [33] in order to reduce false discoveries in metabolomics [34]. Overall, three orthogonal instrumental platforms were used to characterize polar/ionic metabolites from plasma and urine using MSI-CE-MS, as well as total (hydrolyzed) plasma fatty acids (FA) by GC-MS, and inorganic urinary electrolytes by CE with UV detection (Figure S2). Also, 2D scores plots from principal component analysis (PCA) of plasma and creatinine-normalized urine metabolome demonstrated good technical precision from pooled samples used as QC (median CV = 4%–12%) as compared to the biological variance from random/single-spot urine (median CV = 65%–78%) and fasting plasma (median CV = 32%–53%) metabolomes (Figure S3). A batch-correction algorithm was also applied to urine metabolome data to minimize signal drift when using MSI-CE-MS [31], where each run is comprised a serial injection of six individual samples together with a QC. Also, control charts for the recovery standard (F-Phe) provide further evidence of acceptable precision (mean CV < 9%) with few outliers exceeding confidence intervals (Figure S3). A complete list of authenticated metabolites (Table S2) is annotated by their accurate mass and relative migration time (*m*/*z*:RMT) under positive (+) or negative (−) ion mode detection, as well as their most likely molecular formula, mass error, level of identification, and compound name. Unambiguous identification of metabolites associated with contrasting diets was performed by spiking with authentic standards (if available) in conjunction with high-resolution MS/MS, which were compared to reference spectra deposited in public data bases (HMDB); otherwise, spectral annotation was guided by in silico fragmentation [36] using recommended reporting standards for metabolite identification [35,42]. An overview of this metabolomics workflow is outlined in Figure 2, which shows the detection of an unknown protonated molecule [M+H^+^] in plasma, followed by its annotation using high-resolution MS, and subsequent identification (level 1) as proline betaine (ProBet) using MS/MS after comparison to an authentic standard at the same optimum collision energy. Quantification of ProBet using an external calibration curve is also demonstrated, and good technical precision (CV = 13%, *n* = 20) was achieved as shown in a control chart based on the repeated analysis of a QC sample in every run throughout the entire study.

### 3.2. Changes in Dietary Intake and Biomarker Classification

Major changes in self-reported dietary patterns were evident after two weeks of assigned diets as summarized in Table 1. Although there were no significant changes in BMI or average caloric intake between the two treatment arms, greater palatability and satiety was previously reported for DIGEST participants assigned to a Prudent diet [25]. As expected, the Prudent diet sub-group (W-P) reported a higher daily intake of dietary fiber (total, insoluble, soluble), major electrolytes (K^+^, Mg^2+^), fruits and/or vegetables, vitamins (A, C, and E), poly:sat, protein, and sugar or total carbohydrates. In contrast, the Western diet sub-group (P-W) had a higher daily intake of fat (total, saturated, and *trans*), sodium (Na^+^), and cholesterol. Figure S4 illustrates the relationship among 20 nutrient categories reflecting contrasting diets when using PCA along with a hierarchical cluster analysis (HCA) with 2D heat map. There was strong co-linearity (*r* > ± 0.70) among most nutrient categories with two distinctive clusters reflecting opposing Prudent and Western eating patterns.

Volcano plots (Figure S5) were initially used to evaluate changes in the metabolic phenotype of participants using minimum cut-off thresholds (i.e., mean fold-change or *FC* > 1.3; *p* < 0.05). Overall, contrasting diets generated pronounced changes in a wide range of plasma and urinary metabolites that were largely absent for the same participants at baseline given modest differences in their habitual diets prior to the start of food provisions (Table S1). For instance, 10 plasma and 16 urinary metabolites were differentially expressed in W-P as compared to P-W diet groups, including four metabolites satisfying a Benjamini-Hochberg/FDR adjustment (*q* < 0.05), including ProBet, 3- methylhistidine (Me-His), and two unknown urinary metabolites subsequently identified (level 2) as hydroxypipecolic acid (OH-PCA), and imidazole propionic acid (ImPA). The identification and quantification of Me-His were confirmed in both plasma and urine (Figure S6), whereas several unknown urinary metabolites were tentatively identified (level 1 or 2) based on their characteristic MS/MS spectra, such as OH-PCA (Figure S7), and acesulfame K (ASK; Figure S8). Similarly, targeted analysis of FAME from hydrolyzed plasma extracts using GC-MS (Figure S9) allows for resolution of low abundance *trans* isomers (linoelaidic acid, C18:2n-6*trans*), and saturated fatty acids (myristic acid, C14:0) from abundant dietary fatty acids (linoleic acid, C18:2n-6*cis*). As expected, several circulating fatty acids (Figure S5) were consistently elevated in circulation following a Western diet due to a higher average consumption of total fats as compared to a Prudent diet.

### 3.3. Biomarkers of Contrasting Diets and Correlation with Diet Records

**Figure 3:**
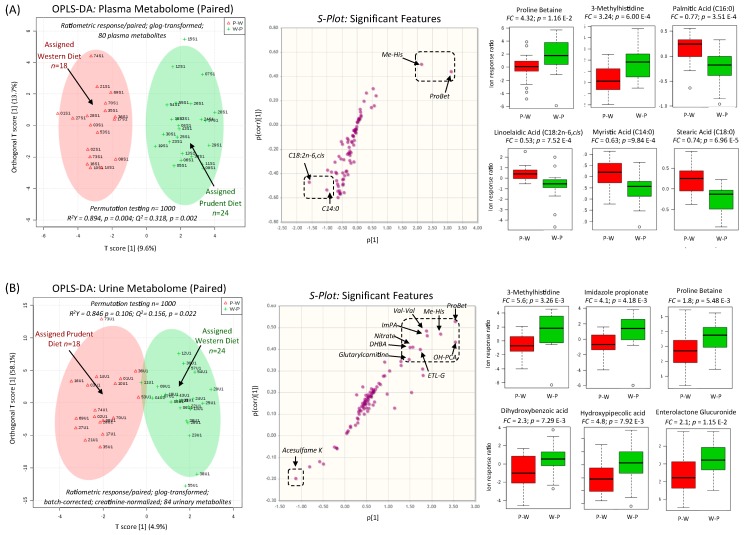
Paired supervised multivariate data analysis of (**A**) plasma and (**B**) creatinine-normalized urine metabolomic data using orthogonal partial least-squares-discriminant analysis (OPLS-DA) using the ratio of ion responses or concentrations for metabolites measured for each participant following two weeks of food provisions relative to their baseline habitual diet. 2D scores plot highlight differences in the overall metabolic phenotype from matching plasma and urine specimens collected from DIGEST participants assigned a Prudent (W-P) or Western (P-W) diet. A sub-set of metabolites responsive to contrasting diets are classified in the S-plots, which were largely consistent with univariate statistical analysis as shown in box-whisker plots for top-ranked metabolites differentially expressed between the two treatment arms (*p* < 0.05).

Complementary statistical methods that take advantage of the repeated-measures study design were used to classify metabolites responses to contrasting dietary patterns. A paired orthogonal partial least-squares discriminant analysis (OPLS-DA) model (Figure 3) was used to initially rank metabolites in plasma and urine that were modulated by contrasting diets relative to each participant’s baseline habitual diet (i.e., ion response ratio). Both OPLS-DA models demonstrated good accuracy (*R^2^* > 0.840) with adequate robustness (*Q^2^* > 0.200) after permutation testing (*p* < 0.05, *n* = 1000). S-plots confirmed that ProBet and Me-His were consistently elevated following a Prudent diet (W-P) in both plasma and urine samples, whereas total plasma C14:0 and C18:2n-6*cis* had the most significant increase following an assigned Western diet (P-W). Additionally, top-ranked creatinine-normalized urinary metabolites excreted at higher levels following a Prudent diet included ImPA, OH-PCA, dihydroxybenzoic acid (DHBA), enterolactone glucuronide (ETL-G), nitrate, and an unknown cation (*m*/*z* 217.156, [M+H^+^]) tentatively identified as a dipeptide, valinyl-valine (Val-Val). In contrast, urinary excretion of ASK was only modestly increased (*p* = 0.0686) following a Western diet. Additionally, excellent discrimination among DIGEST participants following a Prudent or Western diet was achieved when using top-ranked single or ratiometric biomarkers from a receiver operating characteristic (ROC) curve (*AUC* > 0.820; *p* < 1.0 × 10^−5^) for plasma and creatinine-normalized urine samples (Figure S10). For instance, plasma ProBet and the ratio of Me-His/C18:3n-6*trans* demonstrated good sensitivity and specificity (≈ 80–90%) for classifying DIGEST participants based on their assigned diets similar to urinary OH-PCA and the ratio of OH-PCA/Na^+^. A multivariate empirical Bayes analysis of variance (MEBA) [41] was also used to characterize time-dependent metabolite profiles related to contrasting diets after two weeks of food provisions. In this case, metabolic trajectories with distinctive time-course profiles following a Prudent or Western diet were ranked based on their Hotelling’s *T^2^* distribution as shown for plasma (Figure S11) and urine (Figure S12), which were consistent with metabolites identified as dietary biomarkers from volcano plots, ROC curves, and OPLS-DA models.

A mixed ANOVA model, and partial Pearson correlation analysis to self-reported diet records (after adjustments for sex, age, and BMI) were next applied to further validate the relevance of dietary biomarkers identified from multivariate statistical models. Table 2 highlights that ProBet and Me-His were the most robust plasma metabolites associated with a Prudent diet that satisfied several statistical parameters (*T^2^*, *F*-value, *r*, adjusted *p*-value). For instance, ProBet was positively associated (*r* ≈ 0.520, *p* = 0.001) with self-reported mean daily intake of fruit (cup eq./2000 kcal), vitamin C (mg/2000 kcal), and fruits and/or vegetable servings (servings/2000 kcal), as well as negatively associated with fat intake (*r* > −0.530, *p* < 0.001), including *trans* and saturated fat (%energy). Me-His had strong positive correlations (*r* = 0.530−0.570, *p* < 0.001) with average daily intake of protein (%energy), insoluble fiber (g/2000 kcal), electrolytes (Mg^2+^, K^+^; mg/2000 kcal), as well as fruits and/or vegetables reflecting a Prudent diet. Other plasma metabolites classified as dietary biomarkers of contrasting diets included, two carnitines (e.g., carnitine, C0; deoxycarnitine, dC0), two amino acids (e.g., proline, Pro; alanine, Ala), three ketone bodies/intermediates (e.g., ketoleucine; kLeu; ketovaline, kVal; 3-hydroxybutyric acid, OH-BA), and several long-chain fatty acids (e.g., C14:0, C15:0, C18:2n-6*trans*, C18:3n-6*cis*, C18:2n-6*cis*).

Overall, all total (hydrolyzed) plasma fatty acids were positively correlated to a Western diet with a higher average intake of fats (*trans* fats, sat. fats), and a corresponding lower intake of fruits and/or vegetables, poly:sat and micronutrients (vitamins A, C, and E). Similar outcomes were also measured for plasma carnitines and amino acids, which were positively correlated to a Western diet. In contrast, metabolic intermediates of branched-chain amino acids and energy metabolism, namely, plasma kLeu, kVal and OH-BA, were positively associated with a Prudent diet, including higher average intake of protein, fiber, fruits and/or vegetables, poly:sat, and vitamins. Table 2 summarizes 14 plasma metabolites that function as robust biomarkers of contrasting diets since they satisfied at least two of the three statistical models (*p* < 0.05) following adjustment for covariates between groups while also having a significant correlation (*r* > ± 0.3, *p* < 0.05) with at least two nutrient categories from self-reported diet records. An analogous strategy was also used to identify 8 creatinine-normalized urinary metabolites significantly associated with contrasting diets (Table 3). Urinary Me-His and ProBet were among the top-ranked metabolites sensitive to short-term changes in habitual diet with strong positive associations with a Prudent diet. Additionally, several other urinary metabolites were excreted at higher levels for DIGEST participants following a Prudent diet, including OH-PCA and ImPA. Furthermore, two plant-derived phenolic metabolites in urine, namely ETL-G and DHBA were also correlated to healthy eating patterns, such as greater intake of fruits, vegetables and micronutrients. However, creatinine-normalized Val-Val and DMG in urine were weakly correlated with only two nutrient categories (*p* ≈ 0.05) from self-reported diet records. Interestingly, urinary ASK, nitrate and an unidentified cation (*m*/*z*:RMT, 276.144:0.858, [M+H^+^]) were not correlated to any major nutrient from self-reported diet records despite showing treatment responses to contrasting diets.

### 3.4. Metabolic Trajectories and Metabolite Correlation Analysis

**Figure 4:**
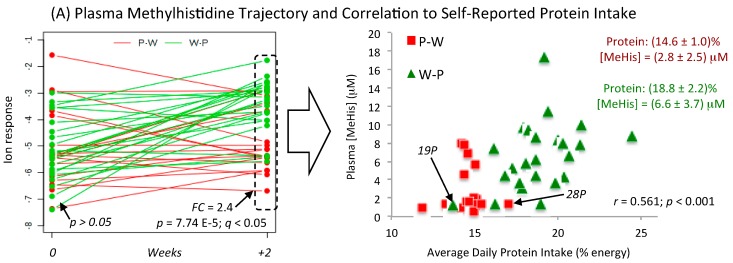

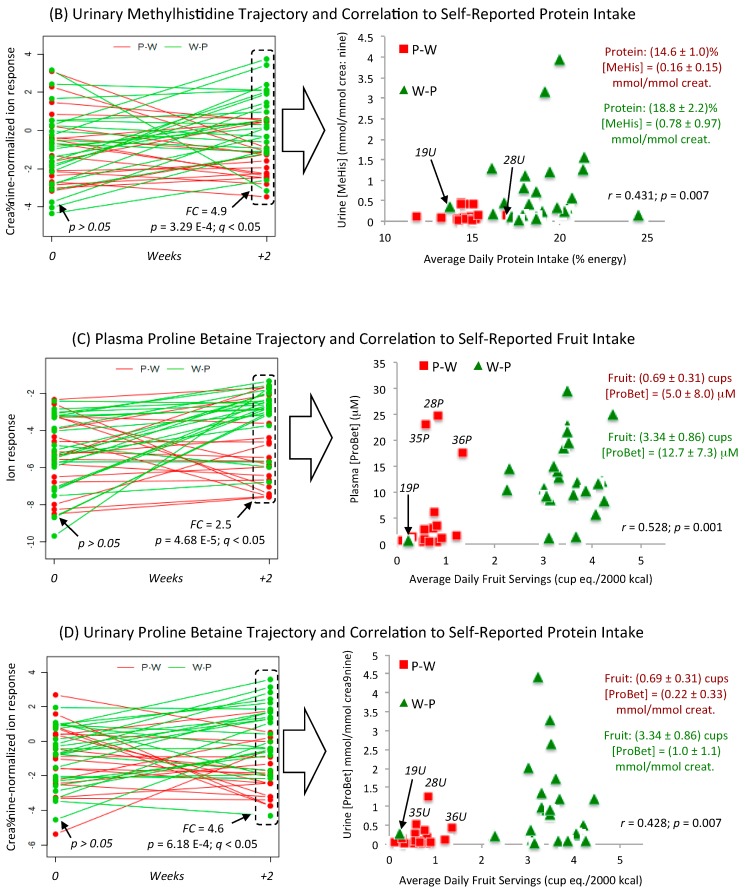
Metabolic trajectories for two dietary biomarkers in plasma and urine that increased following a Prudent diet (W-P) as compared to a Western diet (P-W), namely (**A**,**B**) Me-His and (**C**,**D**) ProBet. Both metabolites were not different at baseline, but undergo significant changes after two weeks of assigned food provisions (*q* < 0.05, FDR) with concentrations moderately correlated (*r* > 0.400) to self-reported diet records, such as a higher average daily intake of fruit (ProBet) and protein (Me-His). Good dietary adherence was demonstrated for most DIGEST participants with only a few exceptions (labeled on plots), who had metabolic phenotypes inconsistent with their assigned diets following two weeks of food provisions.

Representative metabolic trajectories are depicted for top-ranked dietary biomarkers that were measured in plasma (Figure S12) and urine (Figure S13) specimens from DIGEST participants. In all cases, metabolic phenotype changes were evident following two weeks of food provisions with the exception of urinary DHBA, which was the only compound different between assigned diet groups at baseline (*p* = 0.00803). The majority of dietary biomarkers underwent an increase in response for participants consuming a Prudent diet except for circulating fatty acids, two amino acids (Pro, Ala) and two carnitines (C0, dC0) in plasma, which increased following a Western diet. Metabolic trajectory plots also highlight considerable between-subject variances to assigned diets while also identifying outliers among certain individuals due to potential dietary non-adherence and/or inaccurate self-reporting. Figure 4 illustrates metabolic trajectory plots for ProBet and Me-His as they were among the most sensitive biomarkers responsive to contrasting diets, and they were also measured consistently in both plasma and urine samples. Scatter plots show the quantitative relationship between Me-His and ProBet concentrations in plasma as compared to their excreted concentrations in urine with the self-reported average daily intake of protein (%energy), and fruit servings (servings/2000 kcal), respectively. For example, there was a 2.4-fold increase in mean plasma Me-His concentration following two weeks of food provisions that corresponded to a 28% greater intake of dietary protein when comparing Prudent (W-P, *n* = 24) and Western (P-W, *n* = 18) diet groups. Similar results were also evident when comparing creatinine-normalized concentrations of Me-His in urine, which generated a 4.8-fold higher mean concentration in the Prudent relative to Western diet treatment arm. Overall, there was a strong correlation between Me-His concentrations and self-reported dietary protein intake (*r* = 0.430 to 0.560) with few exceptions, such as one participant (W-P, #19) who had consistently low Me-His concentrations in both biofluids consistent with their self-reported protein intake that was characteristic of the Western diet group (P-W); this outcome likely indicates non-adherence to their assigned diet. In contrast, a second participant (P-W, #28) had higher Me-His concentrations in both plasma and urine despite their low self-reported protein intake from diet records that was suggestive of a reporting bias.

Figure 4 also depicts metabolic trajectories for plasma and urinary ProBet concentrations after two weeks of food provisions as compared to their baseline habitual diet along with scatter plots depicting their correlation (*r* = 0.430 to 0.530) to daily fruit servings. Similar to Me-His, the same participant (W-P, #19) had lower ProBet concentrations in both plasma and urine with diet records reflecting a Western diet low in fresh fruit intake despite being assigned to the Prudent diet treatment arm. Additionally, three participants had higher than expected plasma ProBet status (P-W, #28, 35, 36) inconsistent with self-reported diet records; interestingly, ProBet concentrations for window when analyzing complementary biofluids for exogenous dietary biomarkers of recent food intake. Overall, there was a strong positive correlation between Me-His (*r* = 0.638) and ProBet (*r* = 0.547) concentrations measured from matching plasma and urine samples (Figure S13) collected at baseline and following assigned diets (*n* = 84). Additionally, 2D heat maps and correlation matrices for top-ranked plasma (14) and urinary (11) metabolites provide insights into their underlying biochemical relationships, or common dietary sources (Figure S14). As expected, urinary imidazole metabolites derived from histidine, Me-His and ImPA (*r* = 0.956), plasma saturated fatty acids, C14:0 and C15:0 (*r* = 0.873), plasma branched-chain amino acid intermediates, kLeu and kVal (*r* = 0.705), as well as biotransformed plant-derived phenol metabolites in urine, DHBA and ETL-G (*r* = 0.662) were among a group of highly co-linear metabolites correlated to similar nutrient categories from self-reported diet records (Table 2; Table 3).

## 4. Discussion

### 4.1. Contrasting Diets from Food Provisions

Accurate assessment tools of complex dietary patterns are needed to promote human health since sub-optimal diets are responsible for about 20% of preventable deaths from non-communicable diseases worldwide [43]. However, few validated biomarkers exist for routine monitoring of habitual diet [44], such as omega-3 fatty acids [45] and water-insoluble fiber [46]. In this work, a panel of metabolites from plasma and urine was demonstrated to respond to short-term dietary changes when applying a cross-platform metabolomics approach with stringent QC (Figure 1; Figure S2), and a rigorous data workflow for metabolite authentication (Figure 2; Figure S3) [30,31,32]. Since DIGEST participants had poor Prudent diet eating habits, and only modest differences associated with their aggregate Western diet scores (*p* = 0.0037) at baseline (Table S1), we hypothesized that assigning a Prudent diet (W-P) from food provisions would likely induce a more pronounced metabolic phenotype change than a Western diet (P-W); indeed, several top-ranked metabolites (*q* < 0.05, FDR) measured in plasma and urine were largely associated with a Prudent diet as shown in volcano plots (Figure S5). Unlike controlled feeding studies within a laboratory setting, DIGEST participants were provided cooking suggestions with meal plans by a dietician that still allowed for flexibility in food preparations [25]. In this study, short-term dietary changes were found to impact the intake of 20 specific nutrient categories from self-reported diet records (Table 1; Figure S4). For instance, a Prudent diet was consistent with higher consumption of dietary fiber, fruits and/or vegetables, electrolytes and vitamins, but with lower intake of dietary fat, sodium and cholesterol in contrast to a Western diet. To the best of our knowledge, this is the first metabolomics study to investigate the impact of contrasting diets using food provisions. As dietary adherence, potential misreporting, and variations in food preparations represent uncontrolled variables in this study, we aimed to identify metabolites from plasma and urine that can serve as robust biomarkers of changes in habitual diet applicable to a free-living population.

### 4.2. Robust Biomarkers of a Prudent Diet Measured in Both Plasma and Urine

ProBet (Figure 2) and Me-His (Figure S6) were among the most significant metabolites (*q* < 0.05, FDR) associated with a Prudent diet, an eating pattern that promotes good health while contributing to chronic disease prevention [47,48]. In this case, ProBet and Me-His displayed opposing metabolic trajectories in both plasma and urine after two weeks of a Prudent or Western diet with no differences measured at baseline (Figure 4). This was a consistent outcome from univariate (Figure S5) and multivariate (Figure 3) statistical methods after adjustments for covariates (sex, age, BMI), including mixed ANOVA and correlation models (Table 2; Table 3). Indeed, plasma ProBet or the ratio of Me-His/C18:3n-6*cis* provided good discrimination (*AUC* ≈ 0.82 to 0.87, *p* < 3.0 × 10^−5^) of contrasting dietary patterns (Figure S10). Additionally, ProBet and Me-His concentrations in plasma and urine were positively associated (*r* ≈ 0.40–0.60, *p* < 0.001) with eating patterns reflecting a Prudent diet, including a higher daily intake of total fiber, fruits and/or vegetables, protein, vitamins, and electrolytes, along with a lower consumption of *trans* or saturated fats as compared to a Western diet. In fact, ProBet is an exogenous biomarker specific to citrus fruit that has been validated in well-controlled feeding studies [49] since it is not prevalent in most other foods [50]. In fact, ProBet has been replicated in large-scale observational studies as a robust dietary biomarker (*r* ≈ 0.40) of recent citrus fruit/juice intake when compared to standardized FFQ, which can be measured in either blood or urine specimens [18].

Me-His has long been reported as an index of myofibrillar muscle protein turn-over under fasting conditions [51], whereas it also can serve as a biomarker of recent meat consumption (e.g., chicken) with lower plasma concentrations measured in vegetarians as compared to omnivores [52]. Consequently, fasting plasma and creatinine-normalized urinary concentrations of ProBet and Me-His were associated with average daily intake of fruit (servings/2000 kcal) and protein (%energy) as the most likely primary food sources (Figure 4), respectively, which also confirmed good dietary adherence among DIGEST participants with only a few exceptions. For instance, one participant following a Prudent diet (#19, W-P) had consistently lower than expected concentrations of ProBet and Me-His in both plasma and urine samples, which correctly corresponded to their self-reported diet records. In contrast, three participants following a Western diet (#25, 36, 38, P-W) were found to have higher than expected plasma ProBet when compared to their diet record, but this trend was less apparent in their matching urine samples. These observations are likely due to incidental intake of fruit juice or citrus beverages not included with food provisions, which also highlights the different detection windows for dietary biomarkers when relying on a single-spot plasma, and random urine sample in this study [16]. For instance, ingestion of ProBet or orange juice results in a peak concentration in circulation (<1–2 h) that reflects more recent intake as compared to its later excretion in urine (<2–24 h) [53]. Nevertheless, there was a strong linear correlation between circulating and excretory concentrations of ProBet (*r* = 0.638) and Me-His (*r* = 0.547) measured in matching plasma and urine samples collected in this study (Figure S13).

### 4.3. Novel Biomarkers Identified Following a Prudent Diet

Two urinary metabolites were also identified by MS/MS (level 2, Figure S7) as sensitive dietary biomarkers (*q* < 0.05, FDR) reflecting a Prudent diet, namely OH-PCA and ImPA (Table 3). Other potential isomeric/isobaric candidates for these metabolites were ruled out by comparing their MS/MS spectra with those predicted in silico using CFM-ID [37] in the absence of authentic standards for more confident identification (level 1). Their metabolic trajectories (Figure S12) displayed a notable increase (*FC* ≈ 4 to 6, *p* < 0.001) in excretion following a Prudent diet with no differences measured at baseline similar to trends for urinary ProBet and Me-His excretion. ImPA is a normal constituent of human urine derived from the metabolism of histidine [54], which has recently been identified as a product of gut microbiota activity that also regulates insulin sensitivity [55]. This highlights the fact that many dietary biomarkers are not only dependent the intake of foods and host (liver) metabolism, but are also co-metabolized by intestinal microbiota with poorly understood effects on human health. Urinary excretion of ImPA was significantly correlated with fiber, fruits and/or vegetables, and protein intake (*r* ≈ 0.50, *p* ≈ 0.001), which comprise eating patterns consistent with a Prudent diet [47]. Similarly, urinary OH-PCA was found to have a moderate correlation with daily intake of total fiber, fruits and/or vegetables, which was also inversely related to total fat. This data indicates that higher excretion of OH-PCA in urine is likely derived from intake of leguminous plants [56], and/or citrus fruits [57] when following a Prudent diet even though it also represents an endogenous lysine metabolite [58] produced by commensal gut microbiota [59]. Indeed, urinary OH-PCA or its ratio to sodium (OH-PCA/Na^+^) discriminated between DIGEST participants from the two diet treatment arms (Figure S10) with good accuracy (*AUC* ≈ 0.83 to 0.88, *p* < 3.0 × 10^−4^), as well as sensitivity and specificity (≈ 90%).

Two other metabolites derived from edible plant sources were also identified by MS/MS (Figure S8) since they were elevated in urine (*FC* ≈ 2.5 to 3.8) following a Prudent diet as shown by their urinary metabolic trajectory plots (Figure S12), namely ETL-G and DHBA. In the case for ETL-G, a MS/MS spectral match based on three characteristic product ions, including a neutral mass loss corresponding to a glucuronic acid (*m*/*z* 176.032) that is in close agreement with published data [60]. These urinary metabolites were consistently associated (*r* ≈ 0.30-0.40, *p* < 0.05) with fruits, vegetables, vitamin C, and/or total sugar intake, and inversely correlated to total fat (Table 3). ETL-G is a major phytoestrogen from dietary plant lignins, and is excreted in urine as its monoglucuronide conjugate following biotransformation by human intestinal bacteria [61]. Even in controlled feeding studies, there is considerable between-subject variation in the urinary excretion of enterolignin metabolites due to complex interactions with liver and colonic environments [62], which has been reported to possess putative anticancer, antioxidant and/or estrogenic activity [63]. DHBA is a major phenolic acid constituent from most cereals (e.g., wheat, rye) [64], which can serve as a biomarker of dietary fiber intake allowing for differentiation of contrasting low and high (> 48 g/day) fiber diets [65]. In fact, urinary DHBA was the only biomarker differentially excreted at baseline, which likely reflected modest differences in fiber intake (*p* = 0.018, Table S1) between DIGEST participant sub-groups (Figure S5). Urinary Val-Val and DMG were also biomarkers related to a Prudent diet, but had weak correlations with only two nutrients (Table 3), whereas the artificial/low calorie sweetener ASK, and inorganic nitrate were not associated with any nutrients from self-reported diet records (Figure 3). ASK was elevated following a Western diet (Figure 3B), but was rather sporadic with frequent missing data (i.e., below detection limit) since it reflects recent intake of certain sugar-sweetened beverages [66]. In contrast, nitrate exposure has been reported to be mainly from vegetable consumption due to agricultural fertilizer usage [67] that is consistent with its greater excretion in urine following a Prudent diet.

The major circulating ketone body, OH-BA and two branched-chain amino acid intermediates, kVal and kLeu also increased in plasma following a Prudent diet as compared to a Western diet (Table 2). Increases in OH-BA from the liver during ketosis occurs during prolonged fasting or following strenuous exercise [32], as well as abrupt changes in habitual diet, such as adopting a low glycemic index or very low carbohydrate diet [19]. In our work, plasma OH-BA was moderately correlated (*r* ≈ 0.42, *p* < 0.01) to increases in daily intake of fruits and/or vegetables, and poly:sat, as well as being inversely associated with saturated and *trans* fat consumption. Since a Prudent diet is characterized by greater consumption of fiber-rich foods having a lower glycemic index, this may contribute to a mild ketogenic physiological state unlike a Western diet that is characterized by regular consumption of processed foods high in salt and added refined sugar, but low in dietary fiber [47,48]. Indeed, a Prudent diet composed of whole foods elicits fewer adverse health effects with better adherence than highly restrictive ketogenic diets, which has been shown to be effective in regulating insulin sensitivity in type 2 diabetes and pre-diabetic patients [68]. Plasma kVal and kLeu were also positively correlated (*r* ≈ 0.45-0.50, *p* < 0.004) with key nutrients associated with a Prudent diet, including greater daily intake of protein, fruits and/or vegetables, poly:sat, and vitamins. Both plasma metabolites are generated by extra-hepatic branched-chain amino acid transferases prior to oxidative decarboxylation and subsequent utilization as energy substrates within muscle tissue [69]. The metabolism of branched-chain amino acids plays other critical roles in human health, including ammonia detoxification, protein biosynthesis and insulin sensitivity [70] while serving as predictive biomarkers of type 2 diabetes [71]. A correlation matrix/heat map (Figure S14) confirms that plasma kLeu and kVal were strongly co-linear (*r* ≈ 0.70, *p* = 6.8 × 10^−14^) while also being closely associated with OH-BA (*r* ≈ 0.48, *p* = 3.7 × 10^−6^) reflecting common dietary patterns that influence their circulating concentrations. Also, urinary Me-His and ImPA (*r* ≈ 0.96, *p* < 1.0 × 10^−15^), as well as plasma C14:0 and C15:0 (*r* ≈ 0.87, *p* = 8.4 × 10^−15^) were among the most strongly correlated metabolites that are derived from consumption of foods rich in dietary histidine and saturated fats, respectively.

### 4.4. Novel Biomarkers Identified Following a Western Diet

Unlike branched-chain amino acid intermediates, two circulating amino acids, Ala and Pro were associated with greater daily intake of dietary fats (saturated, *trans*, total), and inversely correlated to a Prudent diet due to lower consumption of fruits, vegetables, and protein (Table 2). As a result, their plasma metabolic trajectories increased when DIGEST participants were assigned a Western diet after two weeks (Figure S11). Fasting amino acid concentrations reflect long-term habitual diet rather than recent dietary intake, where Ala has been reported to be inversely associated to plant-based protein diets [72]. This is consistent with outcomes in our study, since plasma Ala was negatively correlated to average daily protein intake (*r* ≈ −0.40, *p* = 0.014). Similar to Ala, plasma Pro was reported to be inversely associated with a Prudent diet as measured in a cross-sectional observational study that was adjusted for age, sex, and BMI [73]. This was consistent with our findings since plasma Pro was inversely related to healthy eating patterns, such as lower intake of fruits and/or vegetables, and higher consumption of processed foods with *trans* fats (Table 2). As expected, plasma Pro was correlated (*r* ≈ 0.46–0.49, *p* < 1.0 × 10^−5^) with circulating levels of Ala, as well as C0 that was indicative of a Western diet (Figure S14). Similar outcomes were also measured for two carnitine metabolites (C0 and dC0) since they had metabolic trajectories that increased for DIGEST participants following a Western diet, which were correlated with greater intake of dietary fat, sodium, and cholesterol (Table 2). Although *de novo* synthesis of C0 is derived from dC0 via lysine metabolism, red meat represents a major dietary source of C0 that is also metabolized by gut microbiota with subsequent host hepatic conversion to generate the thrombosis-promoting metabolite, *N*-trimethylamine oxide (TMAO) [74]; however, plasma or urinary TMAO were not found to be modulated by short-term contrasting diets in our study. In fact, recent studies have shown that anaerobic gut microbiota species can also generate TMAO via its atherogenic intermediate, dC0 due to chronic C0 exposure from the diet [75]. Nevertheless, C0 is still widely promoted as a nutritional supplement and ergogenic aide to improve fatty acid energy metabolism, as well as alleviate muscle injury from strenuous exercise [76]. Lastly, a series of plasma total (hydrolyzed) fatty acids had metabolic trajectories that increased when following a Western diet, which were directly associated with greater intake of total, saturated and *trans* fats, and a lower consumption of poly:sat, vitamins, and fruits and/or vegetables (Table 2). For example, a low abundance circulating *trans* fatty acid, C18:2n-6*trans*, as well as saturated fats (C14:0, C15:0), and omega-6 fatty acids, namely C18:3n-6*cis* and C18:2n-6*cis* were among the most significant plasma FA that increased following two weeks of a Western diet. Indeed, high intake of omega-6 [77] and saturated [78] fatty acids has long been associated with a Western diet that increases systemic inflammation and chronic disease risk. Nevertheless, there remains on-going controversy regarding the optimal dietary fat composition needed to promote cardiometabolic health [79]. Recent clinical trials and observational studies have demonstrated that circulating C14:0, C17:0 and notably C15:0 represent dietary biomarkers of dairy fat intake whose impact on human health may likely be beneficial [80]. In contrast, a greater consumption of processed foods containing vegetable oils rich in C18:2n-6*cis*, and other omega-6 fatty acids is likely a major dietary culprit for CVD prevalence in developed countries [81]. Public health policies have been far more effective in the past decade to reduce dietary *trans* fat intake to less than 1% energy based on WHO recommendations with animal meats/dairy now being more significant than industrial sources from partial hydrogenation of vegetable oils [82]. These trends are consistent with data measured in our work, as fasting plasma concentrations of C18:2n-6*trans* were about 0.34% of its stereoisomer and most abundant FA in circulation, C18:2n-6*cis* (Figure S9).

## 5. Conclusions

In summary, a panel of dietary biomarkers that reflect contrasting Prudent and Western diets were identified based on their distinctive metabolic trajectories measured in paired plasma and urine samples using a cross-platform metabolomics strategy. All DIGEST participants were provided foods for consumption over a two week period while maintaining normal lifestyle habits with no significant changes in their caloric intake, BMI, blood pressure, as well as standard lipid or inflammatory biomarkers as compared to baseline. Me-His and ProBet were the most significant dietary biomarkers associated with a Prudent diet consistently measured in both plasma and urine. Also, urinary ImPA, OH-PCA, ETL-G and DHBA, as well as fasting plasma OH-BA, kVal and kLeu were also positively associated with a Prudent diet. These dietary biomarkers reflect greater consumption of health-promoting foods containing insoluble fiber, protein, essential vitamins/electrolytes, and bioactive phytochemicals with a low glycemic index as compared to highly processed foods in contemporary Western diets. Also, a series of circulating saturated and polyunsaturated fatty acids, as well as plasma Ala, Pro, C0 and dC0 were classified as dietary biomarkers of a Western diet reflecting greater intake of fats, cholesterol and salt, but having lower overall nutrient and fiber quality. Other urinary biomarkers of contrasting diets included ASK, nitrate, DMG and Val-Val, but they did not have strong associations with any specific nutrient categories from self-reported food records. Urine offers a less invasive and more convenient biofluid than blood with a wider detection window for tracking dietary biomarkers in observational studies; however it is more variable when relying on random/single-spot samples due to between-subject differences in hydration status that require normalization. Strengths of this study include the use of complementary statistical methods with appropriate adjustments, access to matching biospecimens and food records from participants, and implementation of a validated metabolomics data workflow for biomarker discovery and authentication with stringent QC. However, there were several study limitations, including the short duration of the dietary intervention, as well as modest sample size involving participants recruited from a single center without strict dietary adherence monitoring, or recording of food preparation methods. Future studies that include multiple time points for biomonitoring of long-term changes in habitual diet with greater study power are recommended. Also, the integration of metabolomics with fecal microbiome data is needed given the important roles of commensal gut microbiota in nutrient generation, and metabolite biotransformation that varies between participants. Also, certain dietary biomarkers tentatively identified in this study require further structural elucidation to confirm their exact stereoisomer configuration. The advent of high throughput and low cost metabolomics technologies allows for a more accurate assessment of diet exposures applicable to large-scale epidemiological and clinical intervention studies. This is urgently needed to validate the utility of dietary biomarkers in nutritional epidemiology to better guide public health policies for the prevention of cardiometabolic and neurodegenerative disorders that increasingly impact an aging population. Overall, our results provide strong corroborating evidence of the utility of food exposure biomarkers to accurately differentiate complex dietary patterns in a free-living population outside a laboratory setting. Future metabolomic studies will focus on assessing maternal nutrition during critical stages of fetal development, and its impact on obesity and metabolic syndrome risk in childhood later in life.

## Figures and Tables

**Table 1:** Major changes in dietary patterns following two weeks of a Prudent or Western diet relative to baseline habitual diet of DIGEST participants (*n* = 42) based on self-reported diet records.

Diet Category ^a^	W-P, *n* = 24	P-W, *n* = 18	*p* for Comparison/Outcome ^b^
Δ Insoluble fiber intake (g/2000 kcal)	(14.0 ± 5.3)	(−5.0 ± 3.5)	*p* = 1.4 × 10^−15^; Greater insol. fiber intake in Prudent arm
Δ Mg intake (mg/2000 kcal)	(189 ± 89)	(−134 ± 70)	*p* = 3.5 × 10^−15^; Greater Mg intake in Prudent arm
Δ Fruits + vegetables intake (servings/2000 kcal/day)	(3.6 ± 1.4)	(−1.8 ± 1.3)	*p* = 7.3 × 10^−15^; Greater fruits + vegetables intake in Prudent arm
Δ Total fiber intake (g/2000 kcal)	(16.6 ± 8.4)	(−13.4 ± 8.1)	*p* = 5.2 × 10^−14^; Greater total fiber intake in Prudent arm
Δ Energy from sat. fat (%)	(−5.4 ± 3.2)	(4.6 ± 2.4)	*p* = 1.8 × 10^−13^; Greater intake of sat. fat in Western arm
Δ K intake (mg/2000 kcal)	(1338 ± 617)	(−854 ± 667)	*p* = 2.5 × 10^−13^; Greater K intake in Prudent arm
Δ Vegetable intake (cup eq./2000 kcal)	(1.8 ± 0.80)	(−0.91 ± 0.92)	*p* = 2.4 × 10^−12^; Greater K intake in Prudent arm
Δ Vitamin E (mg/2000 kcal)	(7.7 ± 5.3)	(−7.0 ± 4.0)	*p* = 5.1 × 10^−12^; Higher intake of vitamin E in Prudent arm
Δ Poly:sat (ratio)	(0.47 ± 0.21)	(−0.14 ± 0.18)	*p* = 8.2 × 10^−12^; Greater intake of Poly:sat in Prudent arm
Δ Vitamin C (mg/2000 kcal)	(149 ± 69)	(−40 ± 54)	*p* = 1.2 × 10^−11^; Higher intake of vitamin C in Prudent arm
Δ Soluble fiber intake (g/2000 kcal)	(3.9 ± 2.1)	(−1.5 ± 1.5)	*p* = 2.3 × 10^−11^; Greater total fiber intake in Prudent arm
Δ Fruit intake (cup eq./2000 kcal)	(1.79 ± 0.93)	(−0.92 ± 0.99)	*p* = 5.9 × 10^−11^; Greater fruits intake in Prudent arm
Δ Energy from fat (%)	(−7.5 ± 5.6)	(5.6 ± 5.6)	*p* = 9.0 × 10^−10^; Greater intake of total fat in Western arm
Δ Na intake (mg/2000 kcal)	(−694 ± 590)	(754 ± 658)	*p* = 6.4 × 10^−9^; Greater Na intake in Western arm
Δ Vitamin A (μg/2000 kcal)	(12,973 ± 56,344)	(−7,847 ± 14,060)	*p* = 1.4 × 10^−7^; Higher intake of vitamin A in Prudent arm
Δ Energy from sugar (%)	(8.9 ± 5.4)	(−1.5 ± 5.8)	*p* = 7.3 × 10^−7^; Higher sugar intake in Prudent arm
Δ Energy from protein (%)	(1.9 ± 3.6)	(−3.2 ± 2.7)	*p* = 1.5 × 10^−5^; Greater intake of protein in Prudent arm
Δ Energy from carbohydrates (%)	(8.5 ± 7.8)	(−0.35 ± 5.7)	*p* = 2.9 × 10^−4^; Greater intake of total carbs in Prudent arm
Δ Cholesterol ^b^ (mg/2000 kcal)	(−101 ± 140)	(54 ± 110)	*p* = 4.8 × 10^−4^; Greater intake of cholesterol in Western arm
Δ Energy from *trans* fat (%)	(−0.26 ± 0.55)	(0.27 ± 0.23)	*p* = 6.4 × 10^−4^; Greater intake of *trans* fats in Western arm

^a^ Mean daily differences (Δ) in self-reported dietary patterns were evaluated from food records collected twice over a two week period at clinical visits as compared to the baseline habitual diet of each participant. ^b^ There were no changes in measured total, LDL, and HDL cholesterol based on standard clinical blood measurements when using a two-tailed student’s t-test with equal variance.

**Table 2:** Top-ranked plasma metabolites associated with contrasting diets by DIGEST participants (*n* = 42) when using time series MEBA, mixed ANOVA, and partial correlation analysis.

Metabolite/ID	Identifier	*T* ^2^ ^a^	*F*-value ^b^	*p*-value ^b^	*R* ^c^	*p*-value ^c^	Food Record ^d^
Proline betaine (ProBet) HMDB04827	144.102:0.984 (+) MSI-CE-MS C_7_H_13_NO_2_ Level 1	24.6	8.7	0.007	−0.601 −0.544 −0.528 0.528 0.518	<0.001 <0.001 0.001 0.001 0.001	Change %fat *trans* fat %energy Sat. fat %energy Fruits; Vitamin C Fruits + Vegetables
3-Methylhistidine (Me-His) HMDB00479	170.092:0.664 (+) MSI-CE-MS C_7_H_11_N_2_O_3_ Level 1	24.9	14.0	0.001	0.573 0.561 0.553 0.546 0.534	<0.001 <0.001 <0.001 <0.001 0.001	Magnesium Protein %energy Insoluble Fiber Potassium Fiber; Fruits + Vegetables
Proline (Pro) HMDB00162	116.070:0.927 (+) MSI-CE-MS C_5_H_9_NO_2_ Level 1	14.6	5.9	0.020	0.495 −0.412 −0.378 −0.362 −0.359	0.002 0.010 0.019 0.026 0.027	*trans* fat %energy Fruits + Vegetables Vegetables Fruits Protein %energy
Carnitine (C0) HMDB00062	162.112:0.735 (+) MSI-CE-MS C_7_H_15_NO_3_ Level 1	12.2	8.9	0.005	−0.464 0.426 −0.404 −0.386 −0.368	0.003 0.008 0.012 0.017 0.023	Poly:sat *trans* fat %energy Fruits + Vegetables Vitamin E Vitamin C
Deoxycarnitine or γ-Butyrobetaine (dC0) HMDB01161	146.128:0.700 (+) MSI-CE-MS C_7_H_16_NO_2_ Level 1	11.9	7.9	0.008	0.367 0.366 −0.352 0.340 −0.336	0.024 0.024 0.030 0.037 0.039	Change %fat Cholesterol Magnesium Sodium Poly:sat
Linoelaidic acid (C18:2n-6*trans*) HMDB06270	294/67.1:15.289 GC-MS C_18_H_32_O_2_ Level 1	10.3	21.5	<0.001	−0.579 −0.555 −0.486 0.485 0.464	<0.001 <0.001 0.002 0.002 0.003	Poly:sat Fruits + Vegetables Vitamin C Sat. fat %energy *trans* fat %energy
Pentadecanoic acid (C15:0) HMDB000673	294/67.1:14.171 GC-MS C_18_H_32_O_2_ Level 1	9.9	16.8	<0.001	−0.471 0.408 −0.403 −0.379 0.379	0.003 0.011 0.012 0.019 0.019	Poly:sat Change %fat Fruits + Vegetables Vitamin A Change %sat. fat
Alanine (Ala) HMDB00161	90.056:0.783 (+) MSI-CE-MS C_3_H_7_NO_2_ Level 1	9.6	6.2	0.018	0.452 0.439 0.428 −0.395 0.386	0.004 0.006 0.007 0.014 0.017	Change %sat. fat Change %fat *trans* fat %energy Protein %energy Sat. fat %energy
Ketoleucine or 4-Methyl-2-oxopentanoic acid (kLeu) HMDB00695	129.056:1.209 (−) MSI-CE-MS C_6_H_10_O_3_ Level 1	7.7	4.4	0.043	0.493 −0.459 0.456 0.453 0.452	0.002 0.004 0.004 0.004 0.004	Fruits + Vegetables Sat. fat %energy Fruits Poly:sat Protein %energy
3-Hydroxybutyric acid (OH-BA) HMDB00357	103.040:1.043 (−) MSI-CE-MS C_4_H_8_O_3_ Level 1	7.6	2.9	0.097	0.437 −0.429 0.425 0.419 0.415	0.006 0.007 0.008 0.009 0.01	Fruits *trans* fat %energy Poly:sat Vitamin A Fruits + Vegetables
α-Linoleic acid (C18:3n-6*cis*) HMDB001388	292/79.1:15.096 GC-MS C_18_H_30_O_2_ Level 1	7.0	11.6	0.002	−0.441 −0.397 −0.391 0.391 −0.387	0.006 0.013 0.015 0.015 0.016	Poly:sat Vitamin A Fruits + Vegetables *trans* fat %energy Vitamin E
Ketovaline or α-Isovaleric acid (kVal) HMDB00019	115.040:1.079 (−) MSI-CE-MS C_5_H_8_O_3_ Level 1	6.3	2.4	0.125	0.489 0.472 0.466 0.458 0.451	0.002 0.003 0.003 0.004 0.004	Protein %energy Fiber (total) Fruits + Vegetables Vitamin E Poly:sat
Myristic acid (14:0) HMDB00826	242/74.1:10.336 GC-MS C_15_H_30_O_2_ Level 1	5.0	15.2	<0.001	−0.535 −0.512 0.503 0.465 −0.463	0.001 0.001 0.001 0.003 0.009	Poly:sat Fruits + Vegetables Change %fat Change %sat. fat Vitamin A
Linoleic acid (C18:2n-6*cis*) HMDB000673	294/67.1:14.171 GC-MSC_18_H_32_O_2_ Level 1	2.6	16.4	<0.001	−0.438 0.420 0.412 −0.382 −0.370	0.006 0.009 0.005 0.018 0.022	Poly:sat Change %fat Change %sat. fat Fruits + Vegetables Vitamin A

^a^ Hotelling’s *T*-squared distribution using MEBA on *g*log-transformed metabolomic time series data. ^b^ Mixed ANOVA model derived from within-subject (diet × time interaction, *p* < 0.05) contrasts when adjusted for sex, age, and BMI. ^c^ Partial Pearson correlation of urinary metabolites to food records with listwise deletion when adjusted for sex, age, and BMI, where *r* > ± 0.30 and *p* < 0.05. ^d^ Top five nutrient categories from self-reported food records that were significantly correlated to urinary metabolites following contrasting provisional diets.

**Table 3:** Top-ranked creatinine-normalized metabolites associated with contrasting diets by DIGEST participants (*n* = 42) when using time series MEBA, mixed ANOVA, and partial correlation analysis.

Metabolite/ID	Identifier	*T* ^2^ ^a^	*F*-test ^b^	*p*-value ^b^	*r* ^c^	*p*-value ^c^	Food Record ^d^
3-Methylhistidine (Me-His) HMDB00479	170.092:0.664 (+) MSI-CE-MS C_7_H_11_N_2_O_3_ Level 1	17.9	7.8	0.008	0.524 0.517 0.457 −0.432 0.431	0.001 0.001 0.004 0.007 0.007	Fiber (total) Fruits + Vegetables Vitamin E *trans* fat %energy Protein %energy
5-Hydroxypipecolic acid (OH-PCA) * HMDB0029246	146.081:1.180 (+) MSI-CE-MS C_6_H_11_NO_3_ Level 2	16.3	1.1	0.293	−0.468 0.397 0.390 0.381 0.374	0.003 0.013 0.016 0.018 0.021	Change fat Fiber (total) Fruits + Vegetables Vitamin E Poly:sat
Imidazole propionic acid (ImPA) HMDB02271	141.066:0.690 (+) MSI-CE-MS C_6_H_8_N_2_O_2_ Level 2	16.1	10.8	0.002	0.515 0.511 0.471 0.463 0.444	0.001 0.001 0.003 0.003 0.005	Fiber (total) Fruits + Vegetables Protein %energy Vitamin E Poly:sat
Proline betaine (ProBet) HMDB04827	144.099:0.984 (+) MSI-CE-MS C_7_H_13_NO_2_ Level 1	15.5	10.8	0.002	0.487 −0.487 0.482 0.480 0.469	0.002 0.002 0.002 0.002 0.003	Poly:sat *trans* fat %energy Fiber Fruits + Vegetables Fiber (insoluble)
Valinyl-valine (Val-Val) HMDB0029140	217.156:0.847 (+) MSI-CE-MS C_10_H_20_N_2_O_3_ Level 3	10.9	3.8	0.060	0.320 0.320	0.050 0.050	Poly:sat Vitamin E
Enterolactone glucuronide (ETL-G) HMDB0240377	473.145:0.934 (−) MSI-CE-MS C_24_H_25_O_10_ Level 2	8.0	7.3	0.010	−0.434 0.387 0.340 0.332 0.316	0.006 0.016 0.037 0.042 0.054	Fat Vitamin C Fruits Fruits + Vegetables Vegetables
Dihydroxybenzoic acid (DHBA) * HMDB0001856	153.019:1.576 (−) MSI-CE-MS C_7_H_6_O_4_ Level 2	8.0	7.3	0.010	−0.403 0.383 0.355 0.324 0.310	0.012 0.018 0.029 0.047 0.058	Fat Sugar % energy Vitamin C Vegetables Fruits + Vegetables
Dimethylglycine (DMG) HMDB0000092	104.108:0.569 (+) MSI-CE-MS C_4_H_9_NO_2_ Level 1	2.9	3.6	0.065	0.356 0.322	0.028 0.049	Fruits + Vegetables Fiber (total)

^a^ Hotelling’s *T*-squared distribution using MEBA on *glog*-transformed metabolomic time series data. ^b^ Mixed ANOVA model derived from within-subject (diet × time interaction, *p* < 0.05) contrasts when adjusted for sex, age, and BMI. ^c^ Partial Pearson correlation of urinary metabolites to food records with listwise deletion when adjusted for sex, age, and BMI, where *r* > ± 0.30 and *p* < 0.05. ^d^ Top 5 nutrient categories from self-reported food records that were significantly correlated to urinary metabolites following contrasting provisional diets. * Exact stereochemistry for tentatively identified metabolites (level 2) in urine was uncertain given other potential isomers.

## Supplementary Materials

The following are available online at https://www.mdpi.com/2072-6643/11/10/2407/s1. Table S1: Description of baseline characteristics of DIGEST cohort; Table S2: Summary of authenticated plasma and urinary metabolites; Figure S1: A CONCERT flow diagram illustrating participant selection criteria; Figure S2: Overview of three instrumental platforms used for nontargeted and targeted metabolite profiling; Figure S3: 2D scores plots from PCA summarizing metabolomic data quality with control charts; Figure S4: 2D scores plots from PCA of contrasting Prudent and Western diets including key discriminating nutrients from self-reporting dietary records; Figure S5: volcano plots depicting differentiating plasma and urine metabolites at baseline and following assigned Prudent or Western dietary treatment arms; Figure S6: Data workflow for identification and quantification of Me-His by MSI-CE-MS with QC; Figure S7: Identification of cationic metabolites, OH-PCA, ImPA, and Val-Val by high-resolution MS/MS; Figure S8: Identification of anionic metabolites, ASK, Ent-G, and DHBA by high-resolution MS/MS; Figure S9: Identification of plasma fatty acids, C14:0 and C18:2n-6*cis* by GC-EI-MS; Figure S10: ROC curves for top-ranked single and ratiometric plasma and urinary biomarkers of contrasting Prudent and Western diets; Figure S11: Trajectory plots for plasma metabolites as biomarkers of contrasting diets for individual DIGEST participants; Figure S12: Trajectory plots for creatinine-normalized urinary metabolites as biomarkers of contrasting diets for individual DIGEST participants; Figure S13: Linear correlation plots for plasma and creatinine-normalized concentrations of Me-His and ProBet in plasma and urine for DIGEST participants; Figure S14: 2D heat maps and correlation matrices for plasma and urinary metabolites associated with contrasting diets from food provisions.
