# Supplementary material for: Metabolic Trajectories Following Contrasting Prudent and Western Diets from Food Provisions: Identifying Robust Biomarkers of Short-Term Changes in Habitual Diet
Source: Nutrients. 2019 Oct 9;11(10):2407. doi: 10.3390/nu11102407 (PMC6835357; doi:10.3390/nu11102407)
Supplement: Supplementary file 1 [file nutrients-11-02407-s001.pdf]

SUPPORTING INFORMATION

Nadine Wellington,[1] Meera Shanmuganathan,[1] Russell J. de Souza,[2,3,4] Michael A. Zulyniak,[2,5] Sandi Azab,[1] Jonathon Bloomfield,[1] Alicia Mell,[1] Ritchie Ly,[1] Dipika Desai,[2] Sonia S. Anand,[2,3,4] and Philip Britz-McKibbin[1],*

[1] Department of Chemical and Chemical Biology, McMaster University, Hamilton, ON, L8S 4M1, Canada;

[2] Department of Medicine, McMaster University, Hamilton, ON, L8S 4K1, Canada;

[3] Department of Health Research Methods, Evidence, and Impact, McMaster University, Hamilton, ON, L8S 4K1, Canada;

[4] Population Health Research Institute, Hamilton, ON, L8L 2X2, Canada;

[5] School of Food Science and Nutrition, University of Leeds, Leeds, LS2 9JT, United Kingdom;

* Correspondence: britz@mcmaster.ca (P.B-.M.)

Tables S1-S2; Figures S1-S14

Table S1. Baseline group characteristics of a cohort of healthy participants (*n*=42) recruited in a two-arm parallel randomized clinical trial to compare the effects of a contrasting diet over two weeks (Western or Prudent) from food provisions reflecting changes in habitual dietary patterns.

Variable [a]	Prudent assigned W-P, *n* = 24	Western assigned P-W, *n* = 18	Significance/comments [b]
Sex (n; %)	--	--	More females than males recruited in each arm
F	66%, *n*=16	61%, *n*= 11	
M	33%, *n*= 8	39%, *n*=7	
Age (mean)	(50 ± 18)	(43 ± 20)	*p* = 0.282; Wide disparity in age with no differences between arms
< 50 y	(29 ± 9 *n*=9)	(28 ± 10, *n*=10)	
> 50 y	(62 ± 7, *n*=15)	(63 ± 8, *n*=8)	
BMI (mean)	(28 ± 6)	(26 ± 6)	*p* = 0.164; Wide disparity in body composition and no differences between arms
Lean (19-24.9 kg/m^2)	(23 ± 2, *n*=7)	(22 ± 2, *n*=11)	
Overweight/obese (25-44 kg/m^2)	(30 ± 5, *n*=17)	(31 ± 5, *n*=7)	
Habitual baseline diet index[a]	--	--	*p* = 0.0037; Greater Western habitual dietary patterns in Prudent assigned arm
Prudent diet score	(0.42 ± 0.93)	(0.92 ± 0.68)	
Western diet score	(3.4 ± 0.9)	(0.70 ± 1.31)	
Average caloric intake (kcal)	(1985 ± 560)	(1895 ± 640)	*p* = 0.629; No significant difference in caloric intake
Average fiber intake (/2000 kcal)	(21.3 ± 6.3)	(26.6 ± 8.5)	*p* = 0.018; Higher intake of fiber in Western assigned arm
Average poly:sat fatty acid (ratio)	(0.44 ± 0.16)	(0.58 ± 0.16)	*p* = 0.0067; Higher poly:sat intake in Western assigned arm
Average energy from sat. fat (%)	(11.9 ± 3.2)	(9.8 ± 2.0)	*p* = 0.015; Higher sat. fat intake in Prudent assigned arm
Urinary Na/K (ratio)	(1.31 ± 0.72)	(0.80 ± 0.55)	*p* = 0.016; Higher Na/K intake in Prudent assigned arm
Fasting glucose (mM)	(5.1 ± 1.0)	(4.9 ± 0.4)	*p* = 0.585; No significant difference in fasting glucose
LDL cholesterol (mM)	(3.1 ± 1.0)	(2.8 ± 0.9)	*p* = 0.309; No significant difference in LDL
HDL cholesterol (mM)	(1.55 ± 0.42)	(1.46 ± 0.44)	*p* = 0.693; No significant difference in HDL
Total cholesterol (mM)	(5.2 ± 1.3)	(5.0 ± 1.0)	*p* = 0.621; No significant difference in cholesterol
Triglycerides (mM)	(0.63 ± 0.18)	(0.61 ± 0.18)	*p* = 0.319; No significant difference in triglyerides
ApoB/ApoA1 ratio	(0.60 ± 0.18)	(0.64 ± 0.19)	*p* = 0.430; No significant difference in ApoB/ApoA1
CRP (mg/L)	(3.9 ± 7.0)	(2.3 ± 4.0)	*p* = 0.405; No significant difference in CRP
IL-8 (ng/L)	(9.1 ± 6.5)	(6.8 ± 2.1)	*p* = 0.159; No significant difference in IL-8
Average systolic BP (mmHg)	(121 ± 18)	(114 ± 17)	*p* = 0.461; No significant difference in systolic BP
Average dystolic BP (mmHg)	(78 ± 11)	(74 ± 10)	*p* = 0.372; No significant difference in dystolic BP

[a] *Self-reported diet quality score at baseline was used as a single aggregate index to categorize dietary patterns as predominately Prudent or Western in terms of average daily intake of total fiber, fruits + vegetables, potassium, polyunsaturated:saturated (poly:sat) fatty acid ratio and % saturated fatty acids with a maximum of five points to the scale.* [b] *There were no significant differences (p < 0.05) in classic serum/plasma biomarkers of CVD risk at baseline, as well as following two week dietary intervention between treatment arm among participants in this study.*

Table S2. Summary of authenticated and reliably measured urinary and plasma metabolites detected in the majority of DIGEST participants that were annotated based on their accurate mass (*m/z*), relative migration time (RMT), ionization mode (*p* = ESI+, *n* = ESI-), most likely molecular formula, compound name, confidence level for identification, and technical precision from QC measurements. Overall, there were 70 and 50 polar/ionic metabolites measured in urine (8 electrolytes measured by CE-UV not listed here), and plasma samples by MSI-CE-MS (including recovery standards not listed here), respectively, as well as 26 (total) plasma fatty acids analyzed by GC-MS.

Biofluid	*m/z:RMT:polarity*	*Molecular formula*[a]	*Monoisotopic mass*	*Δm (ppm)*	*Compound ID*	*HMDB ID*	*Level of Confidence*[b]	*%CV*[c]
Urine	76.0757 : 0.546:+	C_3H_9NO	76.0757	0.0	Trimethylamine *N*-oxide	HMDB00925	2	9.86
Urine	104.0706 : 0.667:+	$C_4H_9NO_2$	104.0706	0.0	γ-Aminobutyic acid	HMDB00112	2	39.69
Urine	104.0706 : 0.928:+	$C_4H_9NO_2$	104.0706	0.0	Dimethylglycine	HMDB0000092	2	33.80
Urine	104.1075 : 0.569:+	$C_5H_{14}NO$	104.1075	0.0	Choline	HMDB0000097	2	12.54
Urine	106.0499 : 0.844:+	$C_3H_7NO_3$	106.0498	0.9	Serine	HMDB0000187	2	11.45
Urine	118.0863 : 0.958:+	$C_5H_{11}NO_2$	118.0862	0.8	Betaine	HMDB0000043	2	12.35
Urine	133.0611 : 0.885:+	$C_4H_8N_2O_3$	133.0607	3.0	Asparagine	HMDB0000168	2	12.87
Urine	137.0460 : 1.067:+	$C_5H_4N_4O$	137.0458	1.5	Hypoxanthine	HMDB0000157	2	6.33
Urine	137.0706 : 0.613:+	$C_7H_8N_2O$	137.0709	2.2	Methylnicotinamide	HMDB0003152	2	16.81
Urine	138.0550 : 0.891:+	$C_7H_7NO_2$	138.0549	0.7	*p*-Aminobenzoic acid	HMDB0001392	2	13.31
Urine	141.0660 : 0.690:+	$C_6H_8N_2O_2$	141.0658	1.4	Imidazole propionic acid	HMDB0002820	2	8.23
Urine	144.1020 : 0.967:+	$C_7H_{13}NO_2$	144.1019	0.7	Proline betaine	HMDB0004827	1	12.88
Urine	146.0812 : 1.183:+	$C_6H_{11}NO_3$	146.0811	0.7	Hydroxypipecolic acid	HMDB0029426	2	9.13
Urine	147.0764 : 0.911:+	$C_5H_{10}N_2O_3$	147.0764	0.0	Glutamine	HMDB0000641	2	8.92
Urine	147.1128 : 0.583:+	$C_6H_{14}N_2O_2$	147.1128	0.0	Lysine	HMDB0000182	2	9.50
Urine	148.0604 : 0.924:+	$C_5H_9NO_4$	148.0604	0.0	Glutamic acid	HMDB0003339	2	11.26
Urine	150.0775 : 0.844:+	$C_6H_7N_5$	150.0774	0.7	*Unknown*		4	25.74
Urine	156.0768 : 0.620:+	$C_6H_9N_3O_2$	156.0767	0.6	Histidine	HMDB0000177	2	6.84
Urine	160.0970 : 1.089:+	$C_7H_{13}NO_3$	160.0968	1.2	*Unknown*		2	9.27

Urine	162.1125 : 0.714:+	$C_7H_{15}NO_3$	162.1124	0.6	Carnitine	HMDB0000062	2	9.60
Urine	166.0724 : 0.702:+	$C_6H_7N_5O$	166.0723	0.6	Methylguanine	HMDB0001566	2	8.66
Urine	170.0924 : 0.634:+	$C_7H_{11}N_3O_2$	170.0924	0.0	3-Methylhistidine	HMDB0000479	1	6.14
Urine	175.1190 : 0.602:+	$C_6H_{14}N_4O_2$	175.1189	0.6	Arginine	HMDB0000517	2	8.53
Urine	176.0666 : 0.851:+	$C_5H_9N_3O_4$	176.0666	0.0	Guanidinosuccinic acid	HMDB0003157	2	5.62
Urine	176.1030 : 0.772:+	$C_6H_{13}N_3O_3$	176.1029	0.6	Citrulline	HMDB0000904	2	6.46
Urine	182.0810 : 0.957:+	$C_9H_{11}NO_3$	182.0811	0.5	Tyrosine	HMDB0000158	2	9.86
Urine	189.1598 : 0.603:+	$C_9H_{20}N_2O_2$	189.1597	0.5	Trimethyllysine	HMDB0001325	2	14.16
Urine	204.1230 : 0.758:+	$C_9H_{17}NO_4$	204.123	0.0	Acetylcarnitine	HMDB0000201	2	12.37
Urine	205.0972 : 0.924:+	$C_{11}H_{12}N_2O_2$	205.0971	0.5	Tryptophan	HMDB0000929	2	28.26
Urine	217.1560 : 0.847:+	$C_{10}H_{21}N_2O_3$	217.1546	6.4	Valylvaline	HMDB0029140	2	25.92
Urine	232.1544 : 0.794:+	$C_{11}H_{21}NO_4$	232.1543	0.4	Butyrylcarnitine	HMDB0002013	2	10.77
Urine	260.1495 : 0.817:+	$C_{12}H_{21}NO_5$	260.1492	1.2	*Unknown*		4	16.33
Urine	276.1441 : 0.858:+	$C_{12}H_{21}NO_6$	276.1441	0.0	Glutarylcarnitine	HMDB0013130	2	7.71
Urine	286.2013 : 0.861:+	$C_{15}H_{27}NO_4$	286.2013	0.0	*Unknown*		4	9.81
Urine	367.1509 : 1.084:+	$C_{17}H_{22}N_2O_7$	367.15	2.5	Mannosyltryptophan		2	6.60
Urine	487.2120 : 0.825:+	$C_{18}H_{34}N_2O_{13}$	487.2134	2.9	Glucosylgalactosyl hydroxylysine	HMDB0000585	2	8.54
Urine	89.0244 : 1.534:-	$C_3H_6O_3$	89.0243	1.1	Lactic acid	HMDB0000190	2	27.69
Urine	105.0193 : 1.488:-	$C_3H_6O_4$	105.0193	0.0	Glycerate	HMDB0000139	2	21.65
Urine	121.0295 : 1.142:-	$C_7H_6O_2$	121.0294	0.8	Benzoic acid	HMDB0001870	2	11.25
Urine	124.9914 : 1.663:-	$C_2H_6O_4S$	124.9913	0.8	Ethylsulfate	HMDB0031233	2	14.32
Urine	128.0353 : 1.345:-	$C_5H_7NO_3$	128.0352	0.8	Oxo-proline	HMDB0000267	2	14.51
Urine	135.0299 : 1.306:-	$C_4H_8O_5$	135.0298	0.7	Threonic acid	HMDB62620	2	14.99
Urine	144.0458 : 1.192:-	C_9H_7NO	144.0454	2.8	Indole-3-carboxaldehyde	HMDB0029737	2	19.91

Urine	146.0460 : 1.972:-	$C_5H_9NO_4$	146.0458	1.4	Glutamic acid	HMDB0000148	2	13.12
Urine	153.0193 : 1.576:-	$C_7H_6O_4$	153.0193	0.0	Dihydroxybenzoic acid	HMDB0013677	2	16.22
Urine	161.9869 : 1.561:-	$C_4H_5NO_4S$	161.9866	1.9	Acesulfame K	HMDB0033585	1	25.13
Urine	167.0211 : 1.257:-	$C_5H_4N_4O_3$	167.021	0.6	Uric acid	HMDB0000289	2	12.41
Urine	171.0068 : 1.755:-	$C_3H_9O_6P$	171.0063	2.9	Glycerol phosphate	HMDB0002520	2	8.54
Urine	177.0229 : 1.180:-	$C_6H_{10}O_4S$	177.0226	1.7	*Unknown*		4	26.13
Urine	178.0510 : 1.176:-	$C_9H_9NO_3$	178.0509	0.6	Hippuric acid	HMDB0000714	2	22.57
Urine	181.9917 : 1.463:-	$C_7H_5NO_3S$	181.9917	0.0	Saccharin	HMDB0029723	1	8.38
Urine	182.0459 : 1.221:-	$C_8H_9NO_4$	182.0458	0.5	Pyridoxic acid	HMDB0000017	2	16.91
Urine	185.0820 : 1.781:-	$C_9H_{14}O_4$	185.0819	0.5	*Unknown*		4	18.92
Urine	187.0071 : 1.416:-	$C_7H_8O_4S$	187.007	0.5	*p*-Cresol sulfate	HMDB0011635	2	14.36
Urine	188.9865 : 1.444:-	$C_6H_6O_5S$	188.9862	1.6	Pyrocatechnol sulfate	HMDB0059724	2	32.92
Urine	191.0552 : 1.142:-	$C_7H_{12}O_6$	191.056	4.2	Quinic acid	HMDB0003072	2	13.44
Urine	193.0373 : 1.122:-	$C_7H_6N_4O_3$	193.0366	3.6	*Unknown*		4	13.12
Urine	212.0023 : 1.357:-	$C_8H_7NO_4S$	212.0022	0.5	Indoxyl sulfate	HMDB0000682	2	12.04
Urine	225.0631 : 1.089:-	$C_8H_{10}N_4O_4$	225.0629	0.9	5-Acetylamino-6-formylamino-3-methyluracil	HMDB0011105	2	21.23
Urine	238.0780 : 1.505:-	-	-	-	*Unknown* $[M-2H]^{2-}$		4	14.49
Urine	263.1037 : 1.037:-	$C_{13}H_{16}N_2O_4$	263.1037	0.0	Phenylacetylglutamine	HMDB0006344	2	12.07
Urine	283.0827 : 1.012:-	$C_{13}H_{16}O_7$	283.0823	1.4	*p*-Cresol glucuronide	HMDB0011686	2	13.97
Urine	287.0236 : 1.152:-	$C_{11}H_{12}O_7S$	287.023	2.1	Dihydroxyphenyl-γ-valerolactone sulfate	HMDB0029191	2	26.79
Urine	302.1138 : 1.016:-	$C_{15}H_{17}N_3O_4$	302.1146	2.6	Indoleacetyl glutamine	HMDB0013240	2	7.79
Urine	308.0987 : 0.984:-	$C_{11}H_{19}NO_9$	308.0987	0.0	*Unknown*		4	12.74
Urine	331.1760 : 0.964:-	$C_{17}H_{24}N_4O_3$	331.1776	4.8	*Unknown*		4	10.67
Urine	377.0170 : 1.040:-	$C_{17}H_6N_4O_7$	377.0164	1.6	*Unknown*		4	20.45

Urine	473.1453 : 0.934:-	$C_{24}H_{25}O_{10}$	473.1448	1.1	Enterolactone glucuronide	-	2	14.37
Urine	632.2044 : 0.874:-	$C_{23}H_{39}NO_{19}$	632.2043	0.2	Sialyllactose	HMDB0000825	2	9.78
Urine	112.0515 : 0.710:-	$C_4H_7N_3O$	112.0516	0.9	Creatinine	HMDB0000562	2	9.61
Plasma	76.0402 : 0.732:+	$C_2H_5NO_2$	76.0393	11.8	Glycine	HMDB0000123	2	8.17
Plasma	90.0557 : 0.783:+	$C_3H_7NO_2$	90.0549	8.9	Alanine	HMDB0000161	2	4.15
Plasma	104.1075 : 0.592:+	$C_5H_{14}NO$	104.1075	0.0	Choline	HMDB0000097	2	30.39
Plasma	106.0500 : 0.864:+	$C_3H_7NO_3$	106.0498	1.9	Serine	HMDB0000187	2	3.23
Plasma	114.0662 : 0.635:+	$C_4H_7N_3O$	114.0653	7.9	Creatinine	HMDB0000562	2	32.14
Plasma	116.0705 : 0.927:+	$C_5H_9NO_2$	116.0706	0.9	Proline	HMDB0000162	2	3.55
Plasma	118.0618 : 0.718:+	$C_3H_7N_3O_2$	118.0611	5.9	Guanidoacetic acid	HMDB0000128	2	9.93
Plasma	120.0654 : 0.900:+	$C_4H_9NO_3$	120.0655	0.8	Threonine	HMDB0000167	2	8.14
Plasma	129.0656 : 0.75:+	$C_5H_8N_2O_2$	129.0658	1.5	*Unknown*		3	6.84
Plasma	132.0766 : 0.765:+	$C_4H_9N_3O_2$	132.0767	0.8	Creatine	HMDB0000064	2	4.80
Plasma	132.1017 : 0.873:+	$C_6H_{13}NO_2$	132.1019	1.5	Leucine/isoleucine	HMDB0000687	2	5.20
Plasma	133.0573 : 0.901:+	$C_4H_8N_2O_3$	133.0607	25.6	Asparagine	HMDB0000168	2	4.20
Plasma	137.0459 : 1.066:+	$C_5H_4N_4O$	137.0458	0.7	Hypoxanthine	HMDB0000157	2	12.85
Plasma	144.0988 : 0.984:+	$C_7H_{13}NO_2$	144.1013	17.3	Proline betaine	HMDB0004827	2	45.39
Plasma	146.1182 : 0.699:+	$C_7H_{15}NO_2$	146.1176	4.1	Deoxycarnitine	HMDB0001161	3	26.48
Plasma	147.0761 : 0.922:+	$C_5H_{10}N_2O_3$	147.0763	1.4	Glutamine	HMDB0000641	2	3.81
Plasma	148.0603 : 0.934:+	$C_5H_9NO_4$	148.0603	0.0	Glutamic acid	HMDB0003339	2	8.50
Plasma	150.0583 : 0.910:+	$C_5H_{11}NO_2S$	150.0583	0.0	Methionine	HMDB0000696	2	4.65
Plasma	152.0567 : 0.910:+	$C_5H_5N_5O$	152.0563	2.6	Guanine	HMDB0000132	2	34.61
Plasma	156.0766 : 0.649:+	$C_6H_9N_3O_2$	156.0763	1.9	Histidine	HMDB0000177	2	31.32
Plasma	160.1332 : 0.725:+	$C_8H_{17}NO_2$	160.1323	5.6	2-Aminooctanoic acid	HMDB0000991	2	22.11
Plasma	162.0761 : 0.933:+	$C_6H_{11}NO_4$	162.0753	4.9	Aminoadipic acid	HMDB0000510	2	14.34

Plasma	162.1123 : 0.735:+	$C_7H_{15}NO_3$	162.1123	0.0	Carnitine	HMDB0000062	2	3.89
Plasma	166.086 : 0.9355:+	$C_9H_{11}NO_2$	166.0853	4.2	Phenylalanine	HMDB0000159	2	12.34
Plasma	170.0922 : 0.663:+	$C_7H_{11}N_3O_2$	170.0923	0.6	Methylhistidine	HMDB0000479	2	15.47
Plasma	175.1191 : 0.631:+	$C_6H_{14}N_4O_2$	175.1183	4.6	Arginine	HMDB0000517	2	37.36
Plasma	176.1025 : 0.943:+	$C_6H_{13}N_3O_3$	176.1023	1.1	Citrulline	HMDB0000904	2	4.25
Plasma	182.081 : 0.9616:+	$C_9H_{11}NO_3$	182.0803	3.8	Tyrosine	HMDB0000158	2	3.52
Plasma	189.1337 : 0.635:+	$C_7H_{16}N_4O_2$	189.1343	3.2	Monomethylarginine	HMDB0029416	2	44.31
Plasma	202.1807 : 0.793:+	$C_{11}H_{23}NO_2$	202.1802	2.5	*Unknown*		4	56.73
Plasma	203.1499 : 0.680:+	$C_8H_{18}N_4O_2$	203.1493	3.0	Dimethylarginine	HMDB0003334/ HMDB0001539	2	11.69
Plasma	204.1233 : 0.776:+	$C_9H_{17}NO_4$	204.1223	4.9	Acetylcarnitine	HMDB0000201	2	4.50
Plasma	205.0966 : 0.931:+	$C_{11}H_{12}N_2O_2$	205.0963	1.5	Tryptophan	HMDB0000929	2	17.46
Plasma	241.0289 : 0.950:+	$C_6H_{12}N_2O_4S_2$	241.0303	5.8	Cystine (disulfide)	HMDB0000192	2	5.04
Plasma	247.1441 : 1.146:+	$C_{14}H_{18}N_2O_2$	247.1433	3.2	Tryptophan betaine	HMDB0061115	2	14.53
Plasma	298.0526 : 0.823:+	$C_8H_{15}N_3O_5S_2$	298.0523	1.0	Cysteinylglycine disulfide	HMDB0000709	2	7.87
Plasma	87.0087 : 1.301:-	$C_3H_4O_3$	87.00874	0.5	Pyruvic acid	HMDB0000243	2	14.14
Plasma	89.0244 : 1.136:-	$C_3H_6O_3$	89.02439	0.1	Lactic acid	HMDB0000190	2	8.13
Plasma	103.0400 : 1.019:-	$C_4H_8O_3$	103.04	0.0	3-Hydroxybutyric acid	HMDB0000357	2	6.91
Plasma	103.0400 : 1.043:-	$C_4H_8O_3$	103.04	0.0	2-Hydroxybutyric acid	HMDB0000008	2	11.53
Plasma	115.0400 : 1.078:-	$C_5H_8O_3$	115.04	0.0	Alpha-ketoisovaleric acid	HMDB0000019	2	15.83
Plasma	117.0193 : 1.766:-	$C_4H_6O_4$	117.0193	0.0	Succinic acid	HMDB0000254	2	31.01
Plasma	128.0353 : 1.018:-	$C_5H_7NO_3$	128.0352	0.8	Oxo-proline	HMDB0000267	2	11.92
Plasma	129.0557 : 1.029:-	$C_6H_{10}O_3$	129.0556	0.8	3-methyl-2-oxovaleric acid	HMDB0000491	2	13.47
Plasma	132.0302 : 1.073:-	$C_4H_7NO_4$	132.0302	0.0	Aspartic acid	HMDB0006483	2	12.40
Plasma	133.0142 : 1.783:-	$C_4H_6O_5$	133.0142	0.0	Malic acid	HMDB0000744	2	32.51

Plasma	167.021 : 0.969:-	$C_5H_4N_4O_3$	167.021	0.0	Uric acid	HMDB0000289	2	11.10
Plasma	179.0561 : 0.999:-	$C_6H_{12}O_6$	179.056	0.6	Glucose	HMDB0000122	2	11.73
Plasma	191.0197 : 1.967:-	$C_6H_8O_7$	191.0197	0.0	Citric acid	HMDB0000094	2	21.51
Plasma	195.051 : 0.889:-	$C_6H_{12}O_7$	195.051	0.0	Gluconic acid	HMDB0000625	2	13.37
Plasma	C14:0 : 10.15	$C_{14}H_{28}O_2$	227.2017	-	Myristic acid	HMDB0000806	2	19.55
Plasma	C15:0 : 10.79	$C_{15}H_{30}O_2$	241.2173	-	Pentadecanoic acid	HMDB0000826	2	13.63
Plasma	C16:0 : 11.43	$C_{16}H_{32}O_2$	255.233	-	Palmitic acid	HMDB0000220	2	12.70
Plasma	C16:1 1 : 11.74	$C_{16}H_{30}O_2$	253.2173	-	Hexadecenoic acid	HMDB0037647	2	13.16
Plasma	C16:1 2 : 11.82	$C_{16}H_{30}O_2$	253.2173	-	Palmitoleic acid	HMDB0003229	2	14.46
Plasma	C17:0 : 12.06	$C_{17}H_{34}O_2$	269.2486	-	Margaric acid	HMDB0002259	2	12.27
Plasma	C18:0 : 12.77	$C_{18}H_{36}O_2$	283.2643	-	Stearic acid	HMDB0000827	2	13.24
Plasma	C18:1 1 : 13.20	$C_{18}H_{34}O_2$	281.2486	-	Elaidic acid	HMDB0000573	2	11.85
Plasma	C18:1 2 : 13.26	$C_{18}H_{34}O_2$	281.2486	-	Oleic acid	HMDB0000207	2	8.50
Plasma	C18:2 1 : 13.92	$C_{18}H_{32}O_2$	279.233	-	Linoleic acid	HMDB0000673	2	13.94
Plasma	C18:2 2 : 15.02	$C_{18}H_{32}O_2$	279.233	-	Linoelaidic acid	HMDB0006270	2	33.45
Plasma	C18:3 1 : 14.47	$C_{18}H_{30}O_2$	277.2173	-	γ-Linolenic acid	HMDB0003073	2	17.16
Plasma	C18:3 2 : 14.82	$C_{18}H_{30}O_2$	277.2173	-	α-Linolenic acid	HMDB0001388	2	14.86
Plasma	C20:0 : 14.37	$C_{20}H_{40}O_2$	311.2956	-	Arachidic acid	HMDB0002212	2	13.34
Plasma	C20:1 : 14.88	$C_{20}H_{38}O_2$	309.2799	-	Eicosenoic acid	HMDB0002231	2	30.24
Plasma	C20:2 : 15.75	$C_{20}H_{36}O_2$	307.2643	-	Eicosadienoic acid	HMDB0005060	2	19.19
Plasma	C20:3 1 : 16.41	$C_{20}H_{34}O_2$	305.2486	-	Eicosatrienoic acid	HMDB0002925	2	11.94
Plasma	C20:3 2 : 17.44	$C_{20}H_{34}O_2$	305.2486	-	Unknown		4	18.70
Plasma	C20:4 : 16.94	$C_{20}H_{32}O_2$	303.233	-	Arachidonic acid	HMDB0001043	2	13.71
Plasma	C20:5 : 18.18	$C_{20}H_{30}O_2$	301.2173	-	Eicosapentaenoic acid	HMDB0001999	2	17.27
Plasma	C22:0 : 16.34	$C_{22}H_{44}O_2$	339.3269	-	Behenic acid	HMDB0000944	2	13.57

Plasma	C22:5 1 : 19.43	$C_{22}H_{34}O_2$	329.2486	-	*Unknown*		4	13.76
Plasma	C22:5 2 : 20.8	$C_{22}H_{34}O_2$	329.2486	-	Docosapentaenoic	HMDB0006528	2	14.12
Plasma	C22:6 : 21.34	$C_{22}H_{32}O_2$	327.233	-	Docosahexaenoic acid	HMDB0062579	2	12.88
Plasma	C24:0 : 18.63	$C_{24}H_{48}O_2$	367.3582	-	Lignoceric acid	HMDB0002003	2	12.15
Plasma	C24:1 :19.28	$C_{24}H_{46}O_2$	365.3425	-	Nervonic acid	HMDB0002368	2	10.88

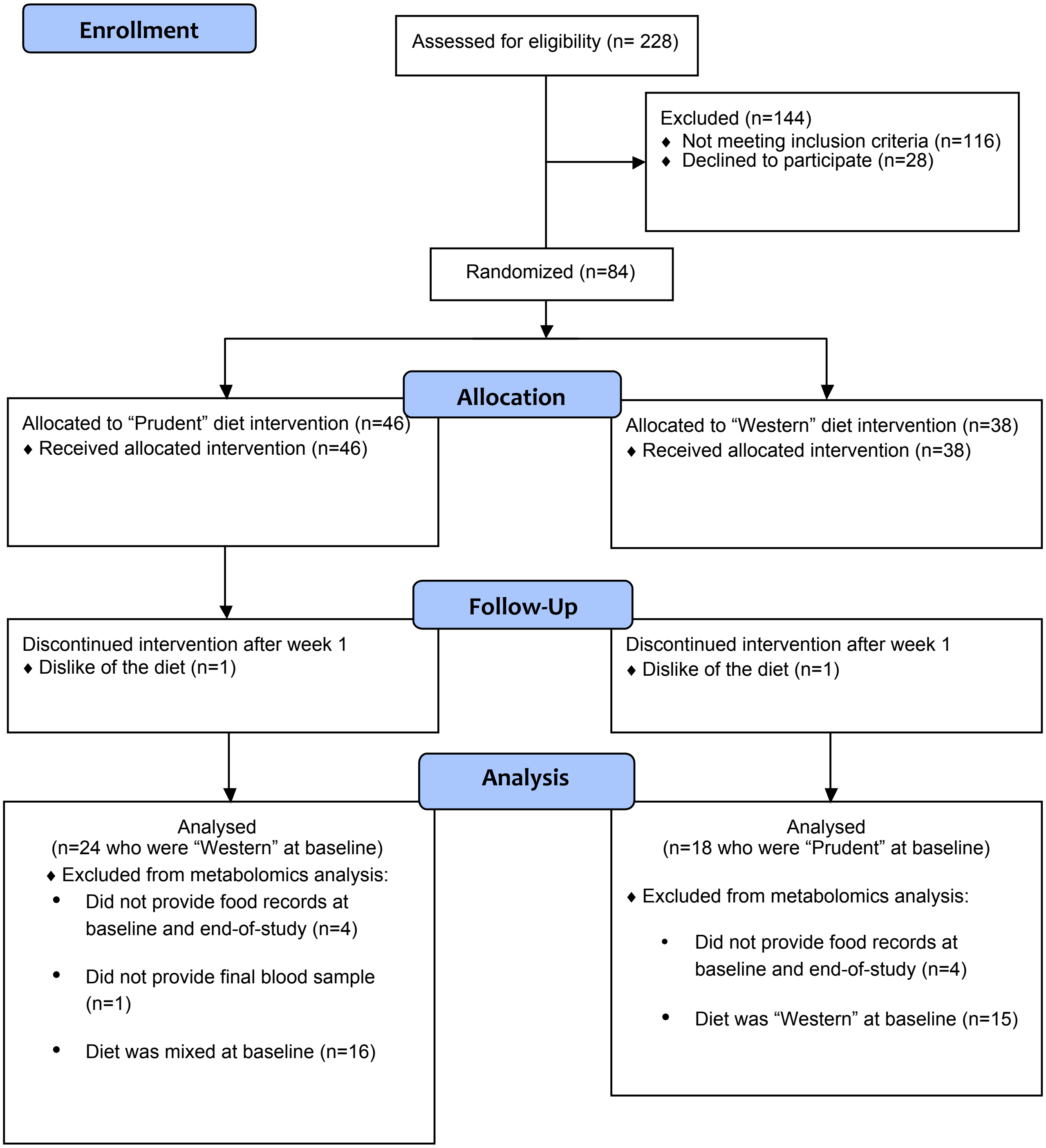

Figure S1. A CONCERT flow diagram outlining selection criteria used in a parallel two-arm randomized clinical trial involving participants from the DIGEST study, where metabolomic analyses was performed unblinded on paired serum and urine samples collected at baseline and two weeks following a provisional Prudent or Western diet. Overall, 73 of the 84 participants who completed DIGEST had available specimens and complete food records. However, in order to maximize the effect size of this short-term dietary intervention, metabolomic analyses was performed only on a subset of participants (n = 42) who had contrasting habitual diets at baseline as evaluated based on an aggregate diet quality score.

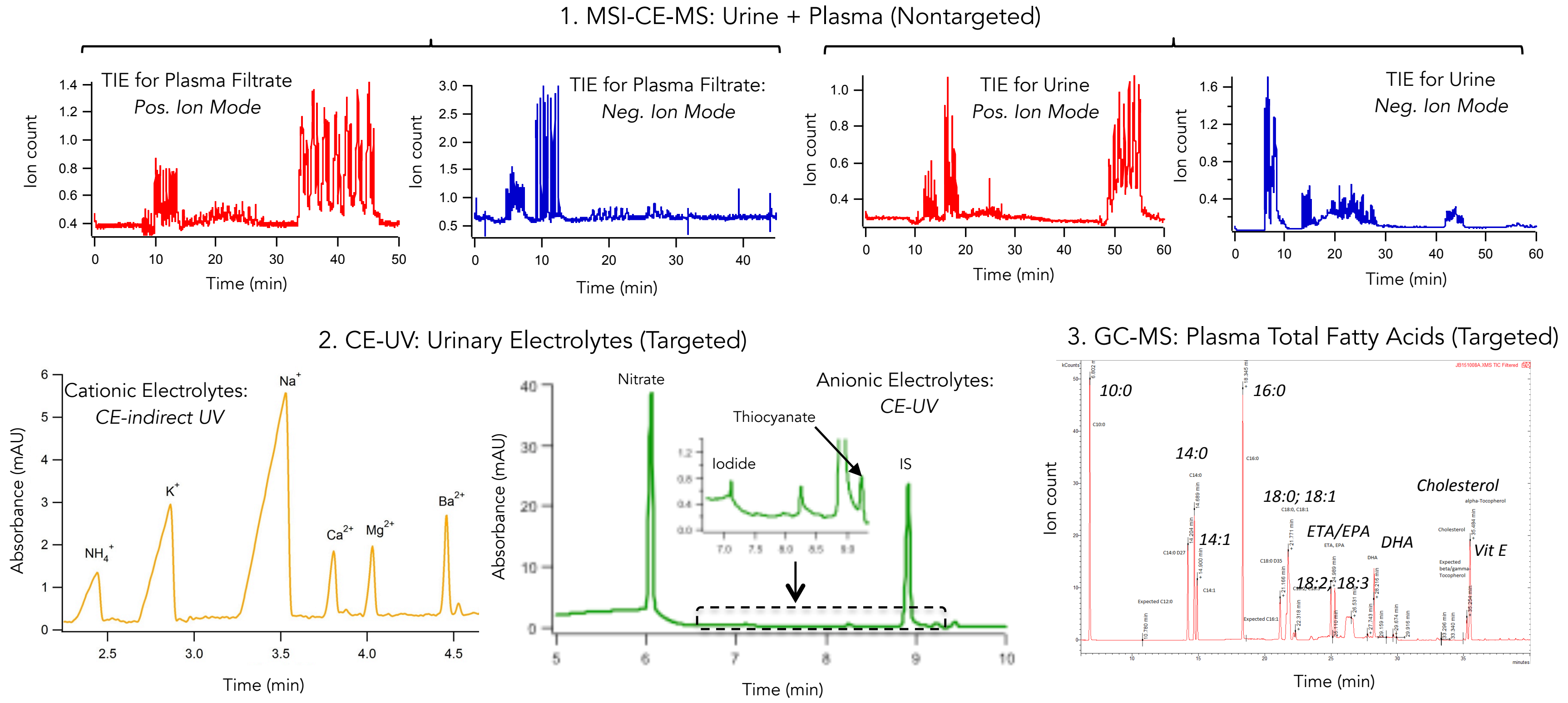

Figure S2. Overview of three major analytical platforms for nontargeted and targeted metabolite/electrolyte profiling of matching plasma and urine specimens from DIGEST participants at baseline and two weeks following an assigned Prudent or Western diet from food provisions. MSI-CE-MS was used as the major format for metabolomic analyses of a wide range of polar/ionic metabolites from both plasma filtrate and diluted urine samples when using a strigent data workflow for metabolite authentication with quality control. Also, CE with (indirect/direct) UV absorbance detection was used for targeted analysis of electrolytes in urine, including inorganic cations (*e.g.,* sodium) and strong anions (*e.g.,* nitrate), whereas GC-MS was applied for targeted analysis of total (hydrolyzed) fatty acids as their FAMEs from plasma extracts.

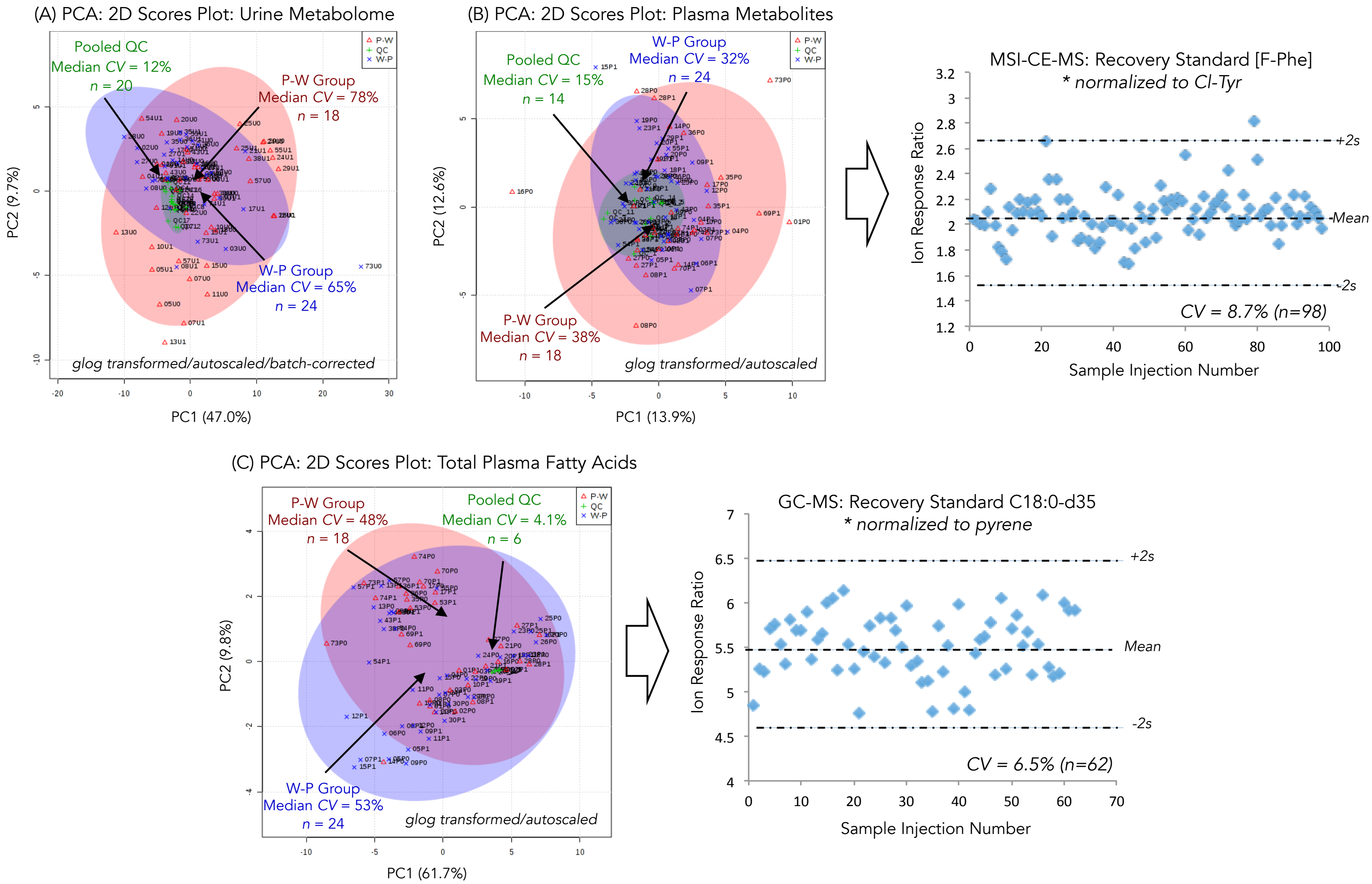

Figure S3. 2D scores plots from PCA and control charts for recovery standards that highlight the good technical precision as compared to biological variance of metabolomic data from three instrumental platforms, including (**A**) 84 authenticated urine metabolites after QC-based batch-correction, (**B**) 50 polar/ionic metabolites from plasma using MSI-CE-MS, as well as (**C**) 26 plasma (total hydrolyzed) fatty acids using GC-MS.

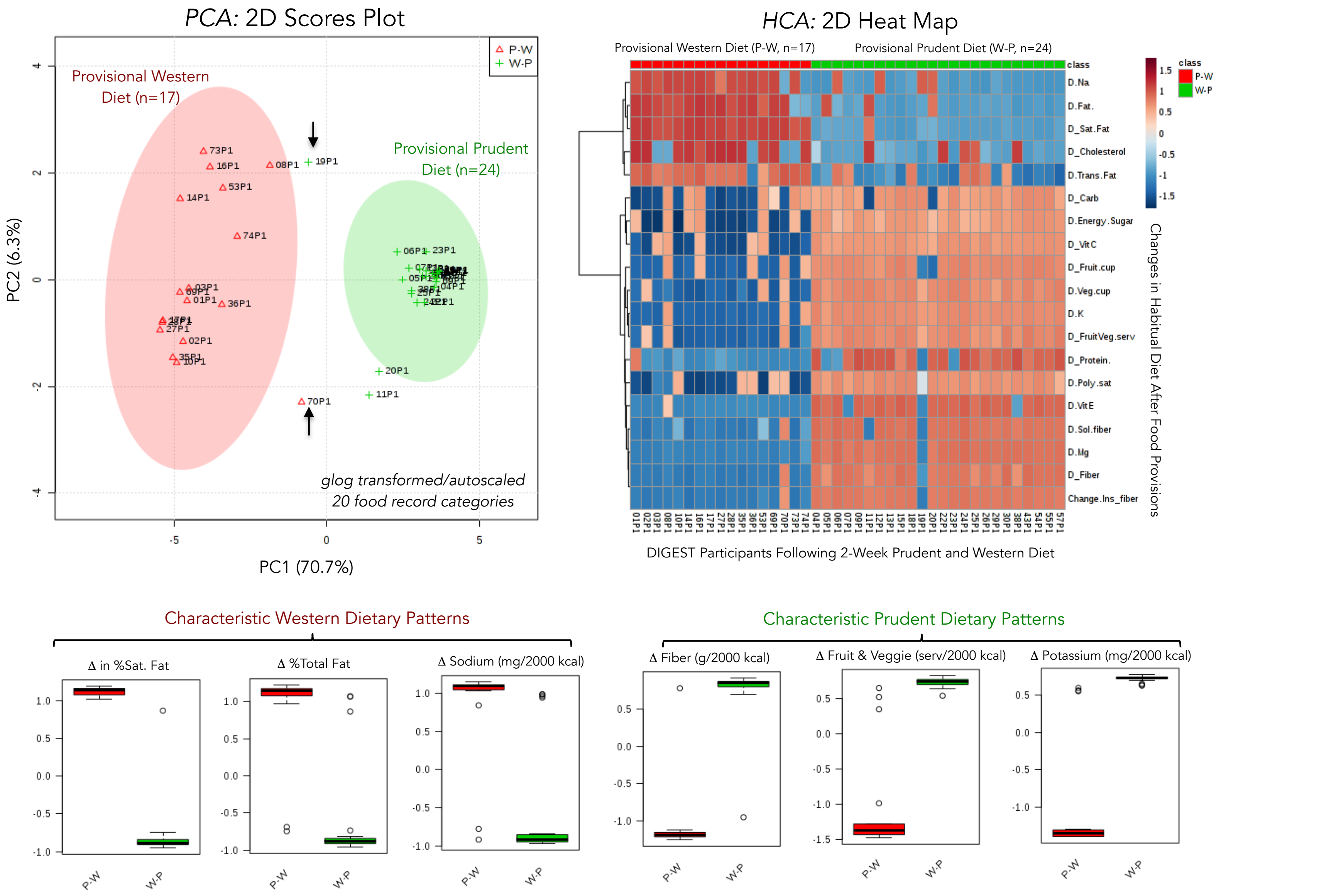

Figure S4. 2D scores plot from PCA and HCA/heat map summarizing changes in habitual diet (W-P and P-W) based on 20 macro-/micronutrient categories self-reported food records from DIGEST participants following two weeks of contrasting food provisions as compared to their baseline diet.

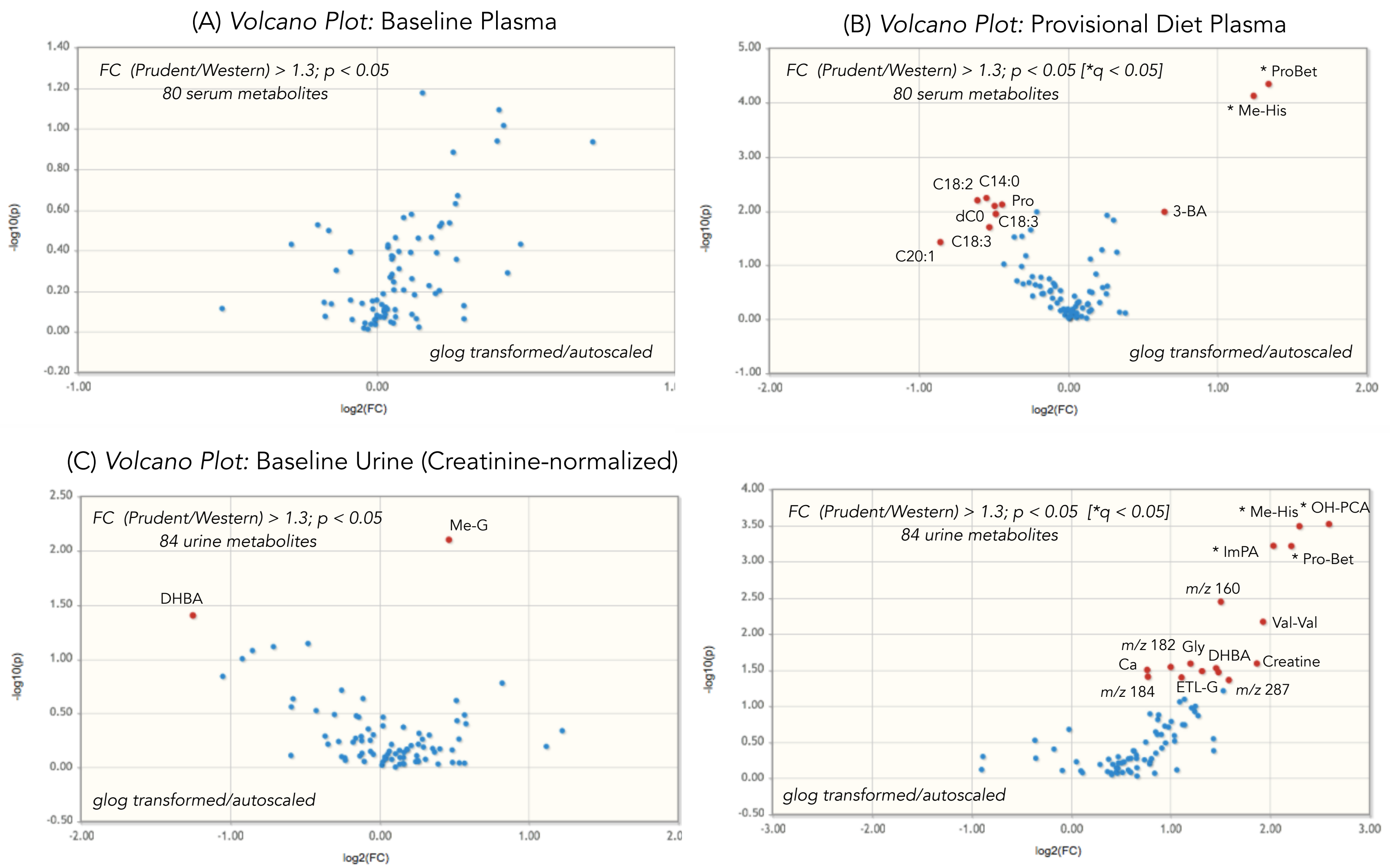

Figure S5. Series of volcano plots for urine (**A**, **C**) and plasma (**B**, **D**) metabolome data highlighting few differences in the metabolic phenotype among DIGEST participants ($n = 42$) at baseline as compared to major changes following two weeks of contrasting diets from food provisions based on a minimum threshold for significance (mean $FC > 1.3$, $p < 0.05$) between Prudent and Western assigned groups (mean $FC > 1.3$ and $p < 0.05$). Abbreviations refer to DHBA (dihydroxybenzoic acid), ProBet (proline betaine), Me-His (3-methylhistidine), 3-OH-BA (3-hydroxybutyric acid), dC0 (deoxycarnitine), Pro (proline), OH-PCA (hydroxypipecolic acid), ImPA (imidazolepropionic acid), ETL-G (enterolactone glucuronide), DHBA (dihydroxybenzoic acid) and Me-G (methylguanine), whereas standard notation are used for plasma fatty acids and unknown ions denoted by their accurate mass (*m/z*).

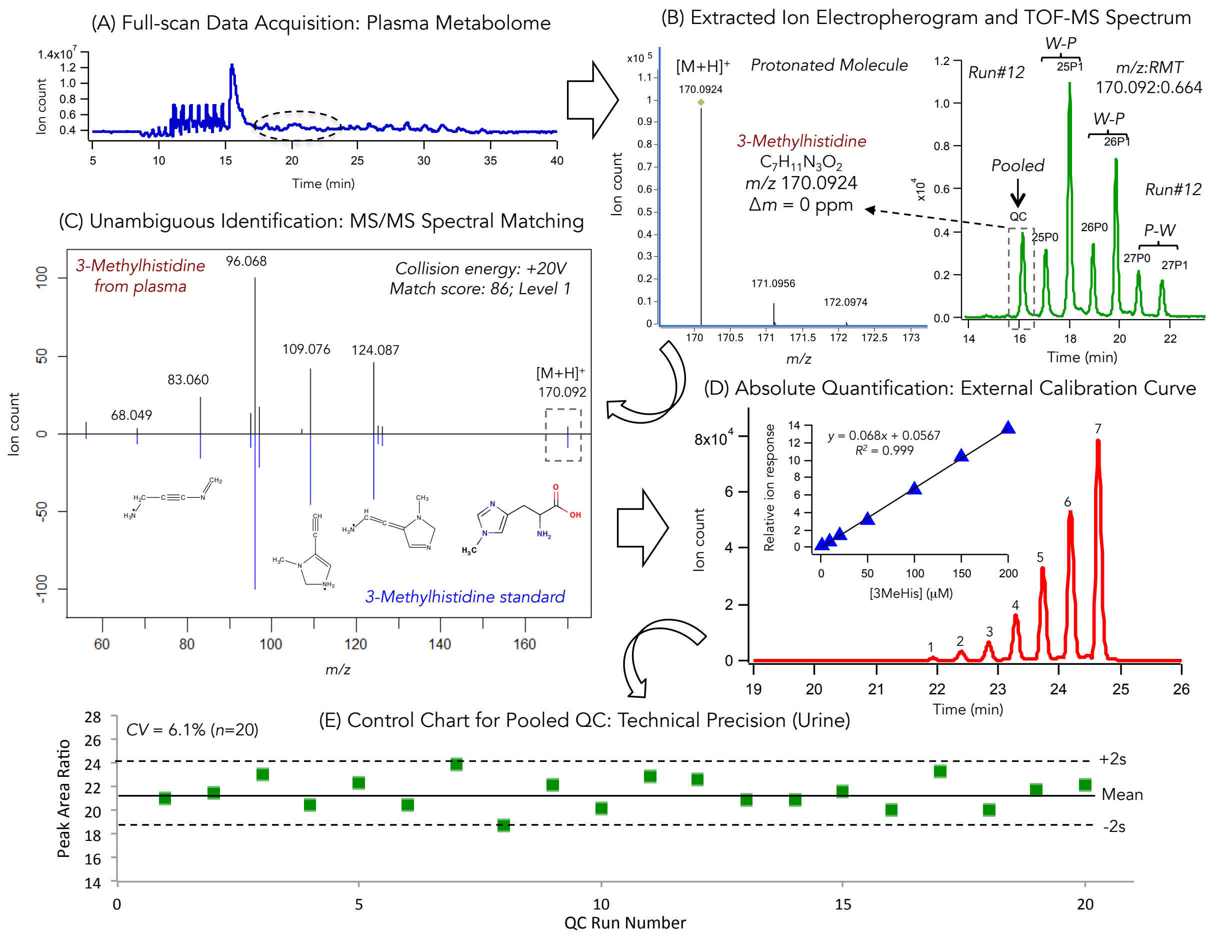

Figure S6. (**A**) Metabolomics data workflow for the identification and quantification of biomarkers of a provisional Prudent diet (*e.g.,* 3-methylhistidine annotated based on its *m/z*:RMT) when using full-scan data acquisition. (**B**) Multiplexed separations by MSI-CE-MS based on serial injection of seven plasma filtrate (or diluted urine) samples within a single run, including paired samples from each DIGEST participant (*i.e.,* baseline/post-treatment) together with a pooled sample as QC for assessing technical precision and long-term signal drift. High-resolution MS under positive ion mode detection allows for determination of most likely molecular formula for unknown cation (*i.e.,* protonated molecular ion), whereas (**C**) MS/MS spectra is used for its structural elucidation when compared with an authentic standard. (**D**) Quantification for metabolites is then performed by external calibration when using an internal standard (Cl-Tyr) for data normalization by MSI-CE-MS. (**E**) A control chart for Me-His from pooled urine samples as QC analyzed in random positions in every run demonstrates acceptable technical precision over 3 days.

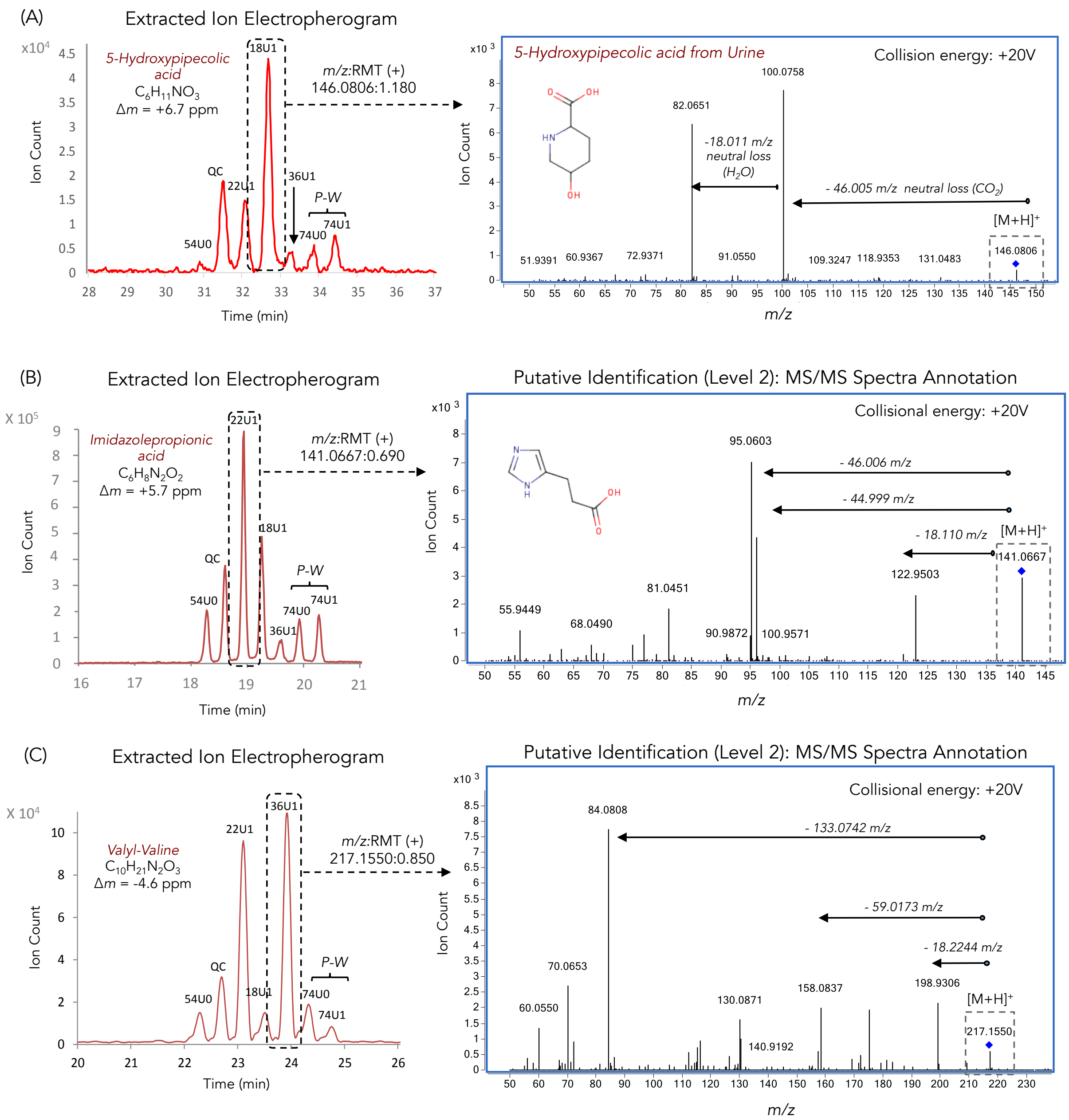

Figure S7. Putative identification (level 2) of three unknown cationic metabolites (MH^+) detected in urine specimens from DIGEST participants that were significantly elevated after an assigned Prudent diet ($p < 0.05$) as compared to Western diet following two weeks of food provisions, namely (**A**) 5-hydroxypipecolic acid (OH-PCA, *m/z* 146.081), (**B**) imidazole propionic acid (ImPA, *m/z* 141.067) and (**C**) Valinyl-valine (Val-Val, *m/z* 217.155) based on their characteristic MS/MS spectra (optimal collision energy at 20 V) under positive ion mode conditions.

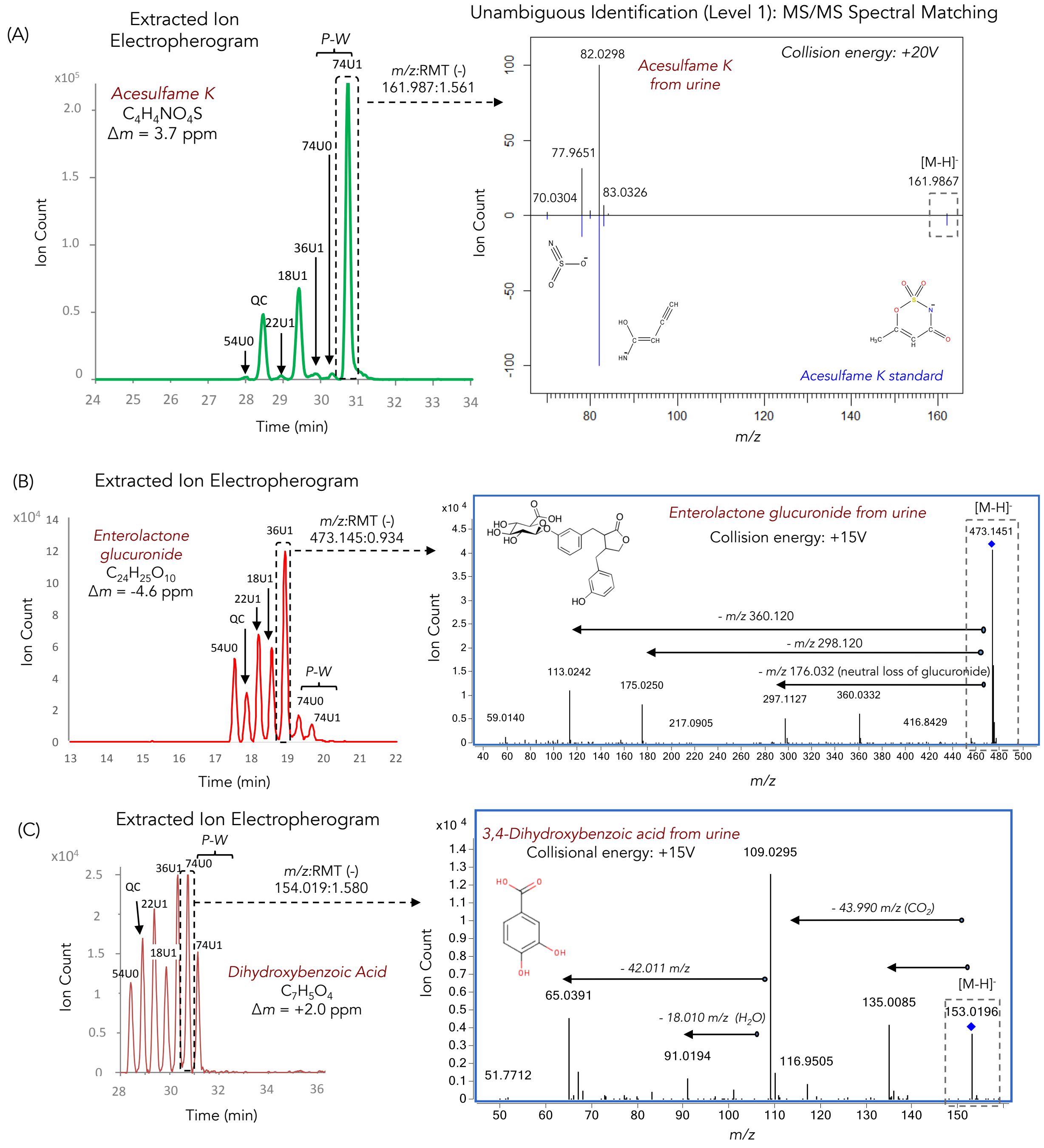

Figure S8. Unambiguous (level 1) and putative identification (level 2) of three unknown anionic metabolites $(M-H^-)$ detected in urine specimens from DIGEST participants that were decreased (acesulfame K) or elevated (enterolactone glucuronide and dihydroxybenzoic acid) in assigned Prudent ($p < 0.05$) as compared to Western diet groups following two weeks of food provisions, namely (**A**) acesulfame K (ASK, *m/z* 161.987), (**B**) enterolactone glucuronide (ETL-G, *m/z* 473.145) and (**C**) dihydroxybenzoic acid (DHBA, *m/z* 153.020) based on their characteristic MS/MS spectra (optimal collision energies at 15 or 20 V) under negative ion mode conditions. Confident identification was derived from comparison of MS/MS spectra with a standard as shown in mirror plot for acesulfame K together with spiking into urine sample to confirm co-migration. The likely stereochemistry for DHBA was deduced from comparison of *in silico* MS/MS spectra when using HMDB, whereas ETL-G was tentatively identified based on comparison with published MS/MS spectra [Johnson et al. *Metabolites* **2013**, 3: 658-672].

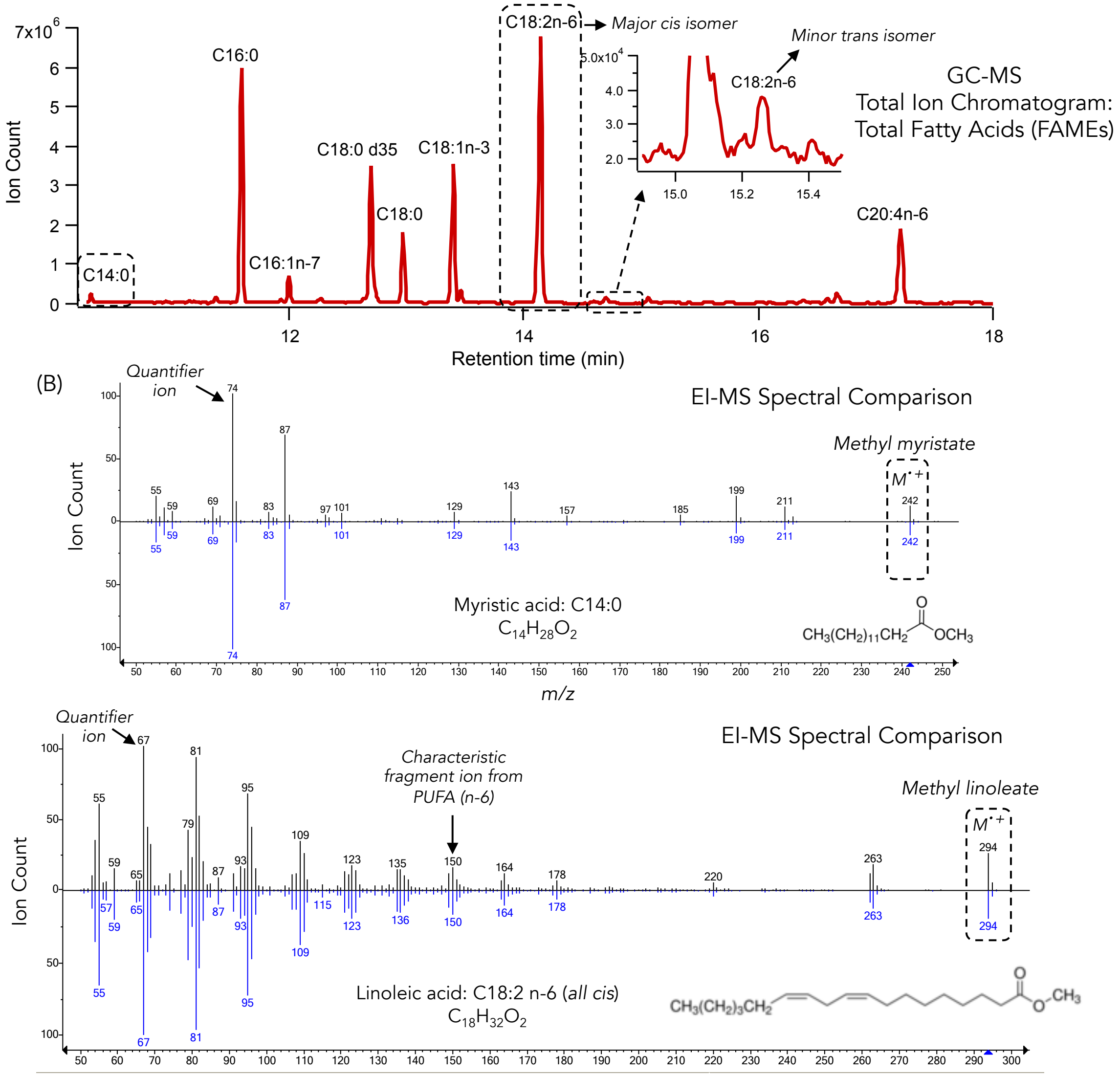

Figure S9. Unambiguous identification (level 1) of total (hydrolyzed) fatty acids as their FAME derivatives from plasma extracts (**A**) when using GC-MS, including resolution (**B**) of low abundance *trans*-isomer (linoelaidic acid, C18:2n-6*trans*) from (**C**) major *cis*-isomer (linoleic acid, C18:2n-6*cis*), including detection of minor saturated fatty acids (myristic acid, C14:0). Mirror plots for EI-MS spectra (**B**,**C**) show excellent matches when comparing FAMEs detected in plasma extracts as compared to their references in the NIST database, where the base peak ions correspond to the quantifier ions monitored for saturated and polyunsaturated fatty acids. Overall, plasma total C18:2n-6*cis* was only 0.34% of its major stereoisomer C18:2n-6*trans* that also represents the most abundant fatty acid measured in circulation.

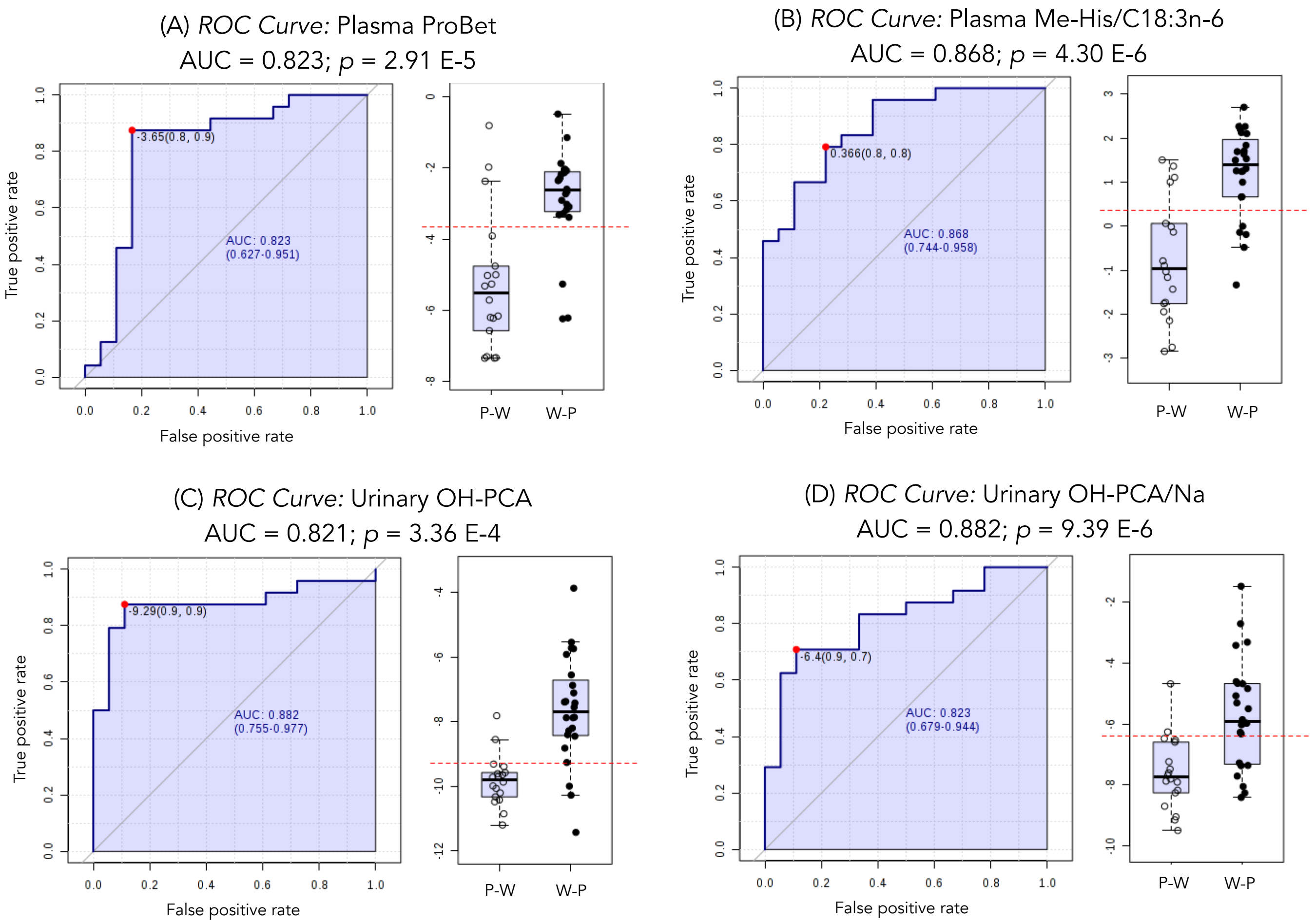

Figure S10. Top-ranked single and ratiometric metabolites for differentiation of DIGEST participants assigned a Prudent (W-P, n = 24) or Western (P-W, n = 18) diet following two weeks of food provisions when using receiver operating characteristic (ROC) curves. All metabolites were *glog*-transformed, whereas urine metabolites were normalized to creatinine following a QC-based batch correction. Overall, there was good discrimination of contrasting dietary patterns ($AUC > 0.820$; $p < 0.001$) as shown for (**A**) plasma proline betaine (ProBet), (**B**) plasma 3-methylhistidine to α-linoleic acid (MeHis/C18:3n-6) ratio, (**C**) urinary hydroxypipecolic acid (OH-PCA), and (**D**) urinary hydroxypipecolic acid to sodium ratio (OH-PCA/Na).

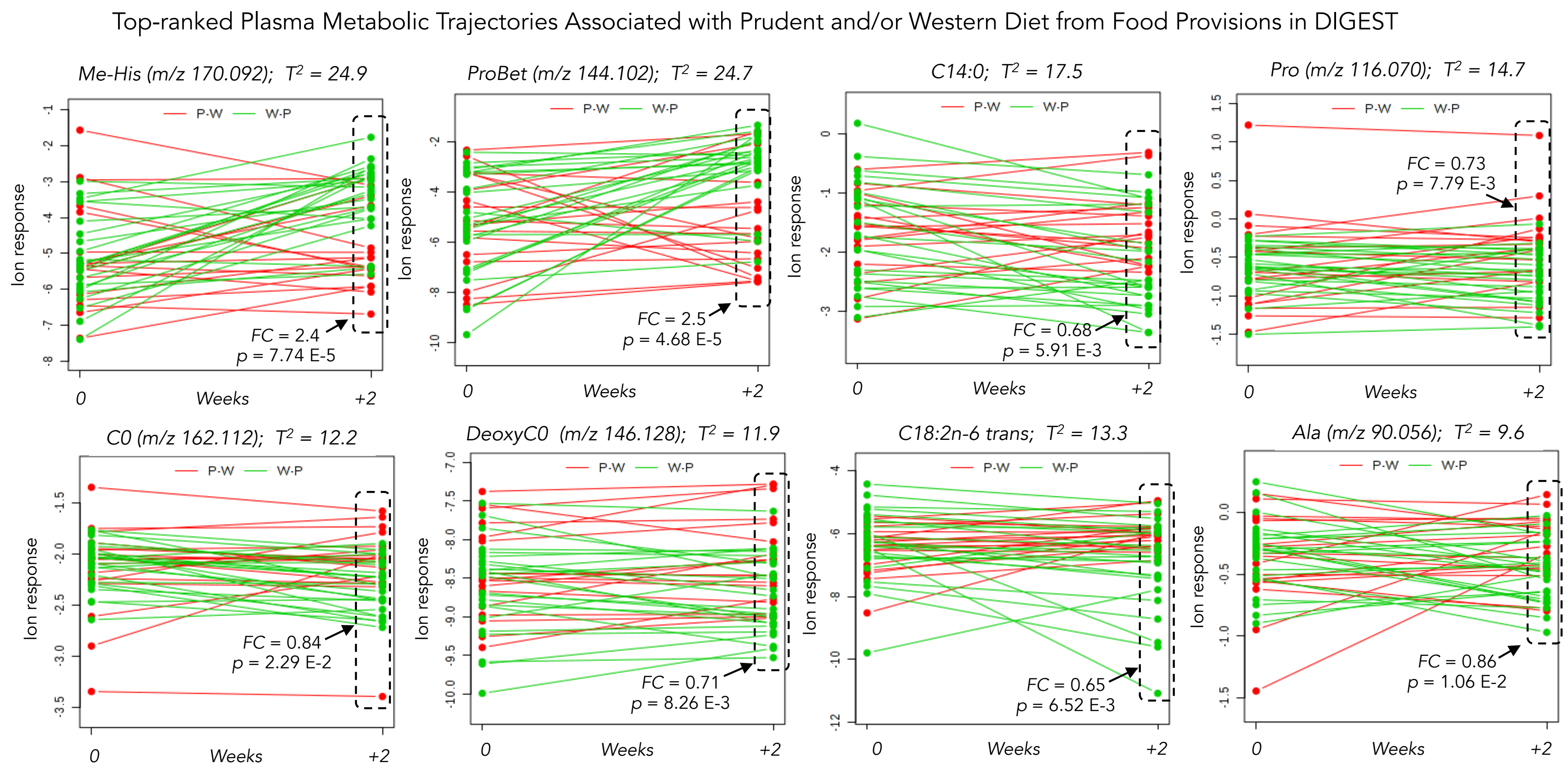

Figure S11. Top-ranked plasma metabolites associated with contrasting Prudent (W-P) or Western (P-W) diets from food provisions when using *glog*-transformed ion responses measured at baseline (habitual diet, 0) and following two weeks of food provisions for DIGEST participants (n = 42). Metabolic trajectories from time course studies were ranked based on Hotelling-T^2 values when using multivariate empirical Bayes analysis of variance (MEBA) together with student's t-test to confirm statistical significance ($p < 0.05$) occurring after assigned diets as compared to baseline habitual diets ($p > 0.05$).

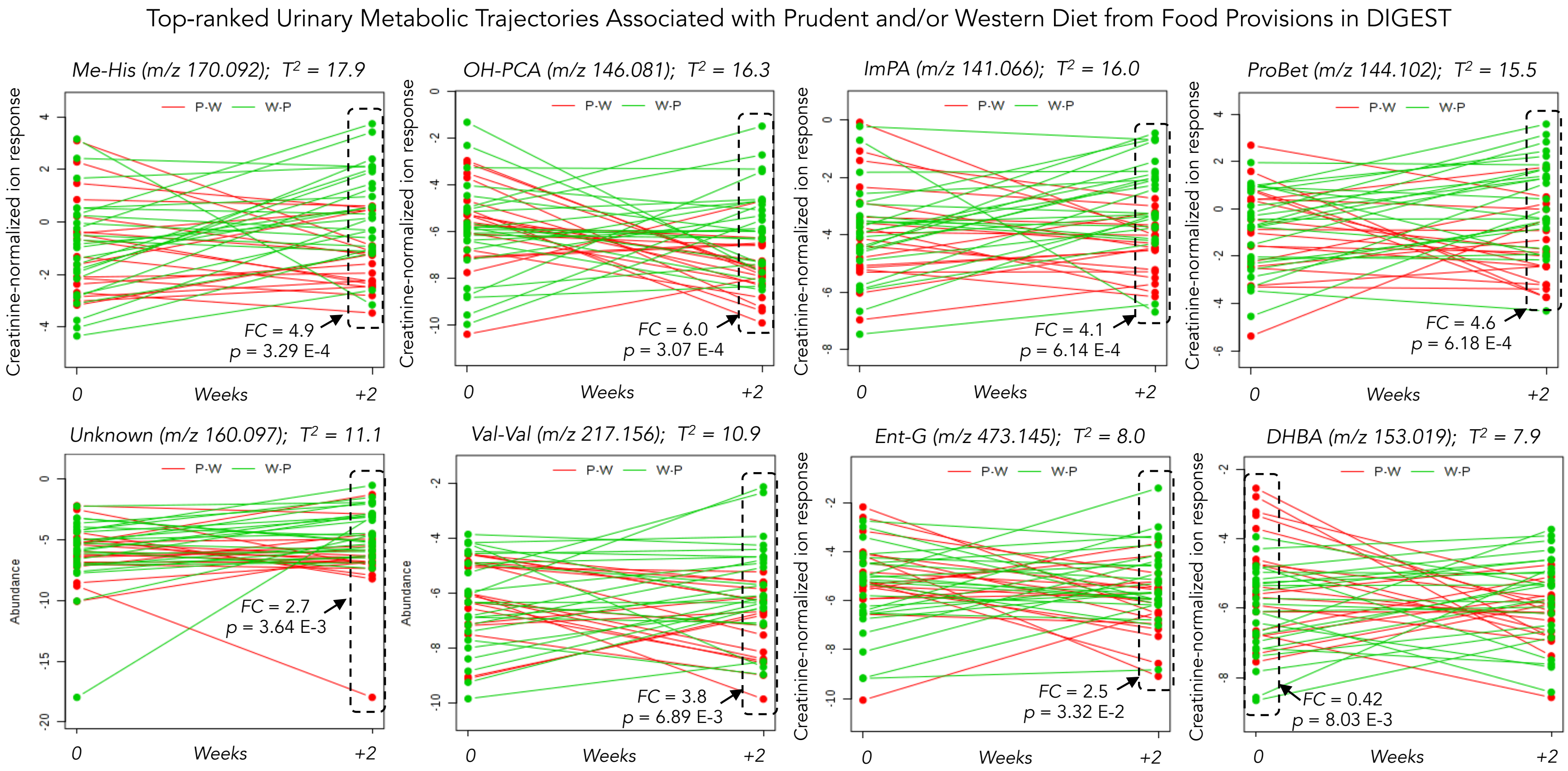

Figure S12. Top-ranked urinary metabolites associated with contrasting Prudent (W-P) or Western (P-W) diets from food provisions when using *glog*-transformed ion responses normalized to creatinine measured at baseline (habitual diet, 0) and following two weeks of food provisions for DIGEST participants ($n = 42$). Metabolic trajectories from time course studies were ranked based on Hotelling-T^2 values when using multivariate empirical Bayes analysis of variance (MEBA) together with student's t-test to confirm statistical significance ($p < 0.05$) occurring after assigned diets as compared to baseline habitual diets ($p > 0.05$) with the exception for DHBA.

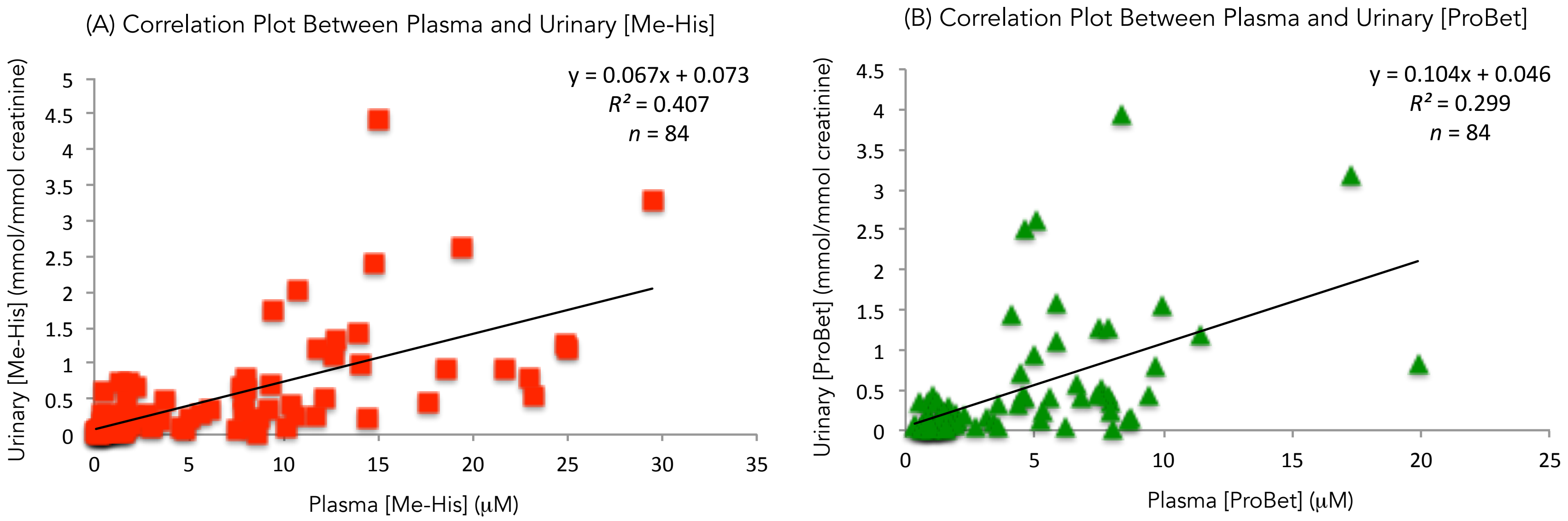

Figure S13. Linear correlation plots highlighting the strong association between fasting plasma concentrations of (**A**) Me-His and (**B**) ProBet and their creatinine-normalized concentrations measured independently from matching single-spot urine samples for DIGEST participants collected at baseline and then following two weeks of food provisions.

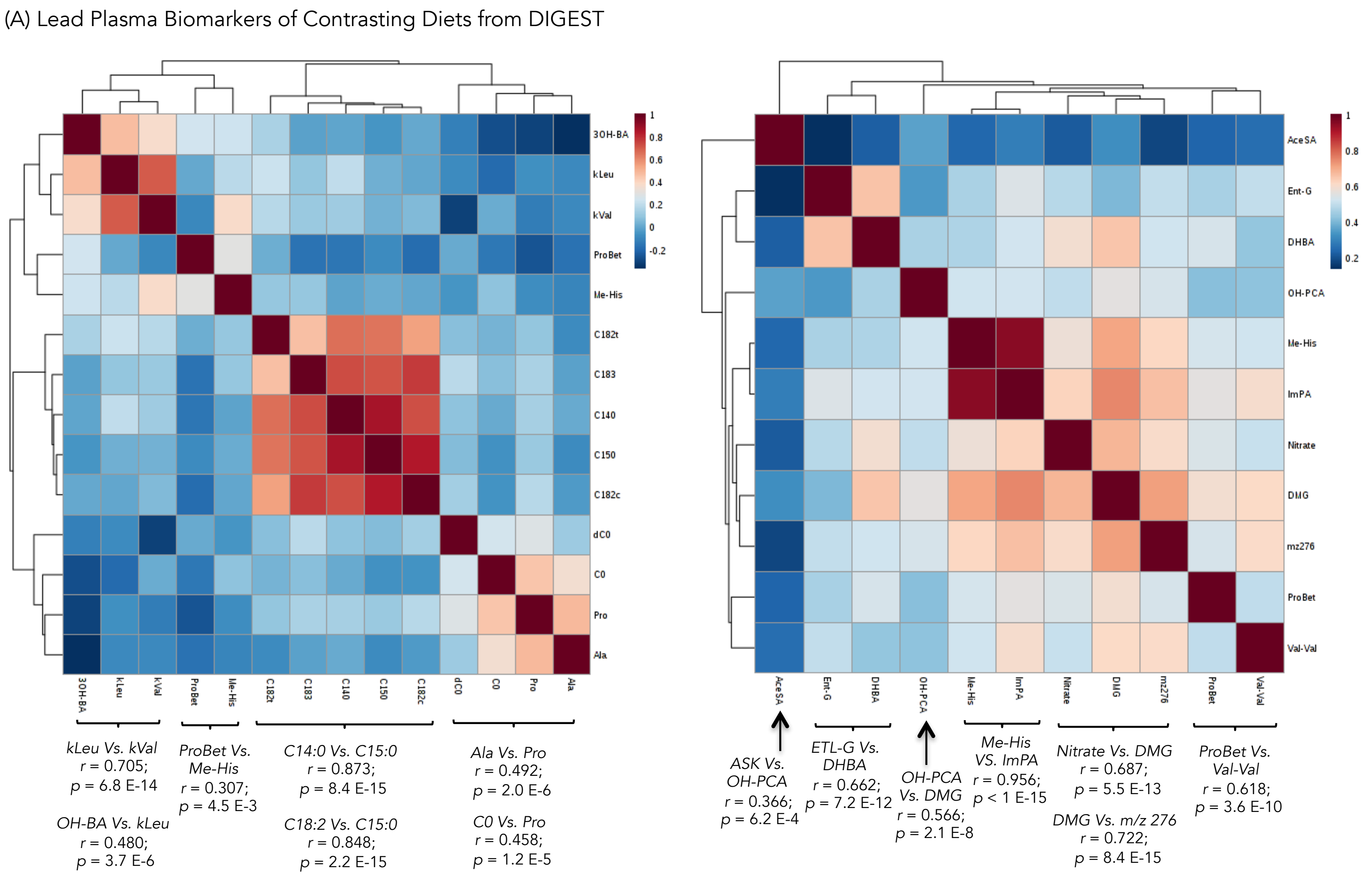

Figure S14. 2D heat maps and correlation matrices for top-ranked (**A**) plasma and (**B**) urinary metabolites associated with contrasting diets from food provisions when using a Pearson correlation analysis on *glog*-transformed data. Distinctive clusters of metabolite classes suggest common dietary sources and/or biochemical pathways for their regulation, such as circulating ketone bodies (kLeu, kVal, 3-OH-BA), fatty acids (C14:0, C15:0, C18:2, C18:3) and amino acids/carnitines (C0, Pro, Ala) in plasma, as well as plant-derived biotransformed phenols (ETL-G, DHBA) and imidazole metabolites (Me-His, ImPA) in urine. In many cases, urinary metabolites reflective of recent dietary patterns were broadly co-linear with other compounds (*e.g.,* OH-PCA, Me-His, ProBet and unknown ion, *m/z* 276) or had modest correlations (*e.g.,* ASK) to other compounds overall.
